# Surface Functionalities of Polymers for Biomaterial Applications

**DOI:** 10.3390/polym14122307

**Published:** 2022-06-07

**Authors:** Mioara Drobota, Stefan Ursache, Magdalena Aflori

**Affiliations:** 1 “Petru Poni” Institute of Macromolecular Chemistry, 41A Aleea Gr. Ghica Voda, 700487 Iasi, Romania; miamiara@icmpp.ro; 2 Innovative Green Power, No. 5 Iancu Bacalu Street, 700029 Iasi, Romania; ursi_stefan@yahoo.com

**Keywords:** biomaterial, surface functionalities, polymers, cell–interface interaction, antimicrobial

## Abstract

Changes of a material biointerface allow for specialized cell signaling and diverse biological responses. Biomaterials incorporating immobilized bioactive ligands have been widely introduced and used for tissue engineering and regenerative medicine applications in order to develop biomaterials with improved functionality. Furthermore, a variety of physical and chemical techniques have been utilized to improve biomaterial functionality, particularly at the material interface. At the interface level, the interactions between materials and cells are described. The importance of surface features in cell function is then examined, with new strategies for surface modification being highlighted in detail.

## 1. Introduction

Many promising therapy options for mending and/or replacing damaged tissues are now available thanks to the rise of tissue engineering and regenerative medicine sectors. Despite the fact that much effort is put into producing biomaterials as viable tissue replacements for clinical use, most of the proposed techniques fail to match the functional features of targeted tissues in vivo due to low biocompatibility. Biocompatibility is determined by the nature of biomaterial interactions with the biological milieu, but there is still a key gap in successfully linking the surface physicochemical features of biomaterials to biochemical signal-transduction pathways. However, it is undeniable that the majority of these approaches rely on chemical principles for developing biomaterials as biological substitutes that replicate and/or promote tissue activities. Surface of the material plays an important role in this context. Surface modification is the process of changing the physical or chemical features of a material’s surface, which can be done at the nanoscale or at the bulk level. Nanoscale surface modification is a key technique in nanotechnology, and it entails nanofabrication that can change the topography and chemistry of a surface at the nanometric level. The alterations of substrates are carried out in the nanodomain in nanoscale modification [1,2,3,4,5,6].

**Figure 1:**
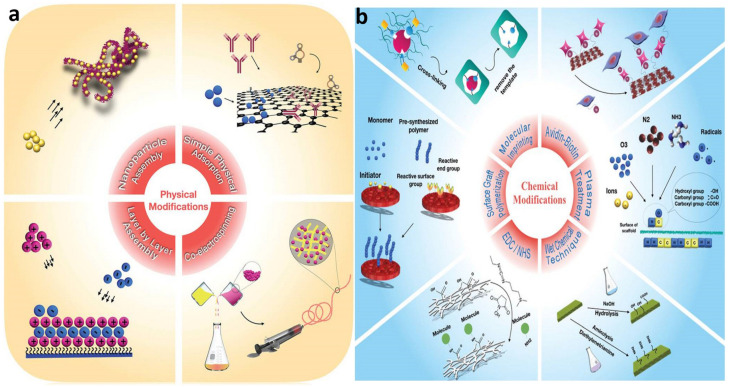
The most representative physical (**a**) and chemical (**b**) modifications of polymers. Reproduced with permission from Ref. [6]. Copyright 2019 WILEY-VCH Verlag GmbH & Co. KGaA, Weinheim, Germany.

While biomaterial design and validation receive a large amount of attention, the nature of their interactions with the surrounding biological milieu is sometimes overlooked. This knowledge gap could be caused by a lack of understanding of biochemical signaling pathways, a lack of dependable procedures for creating biomaterials with appropriate physicochemical qualities, and/or biomaterial properties that are unstable after implantation. The effectiveness of host reactions to biomaterials, known as biocompatibility, is based on chemical concepts that are at the heart of both the body’s cell signaling pathways and the design of the biomaterial surface. Biomedical scientists construct biomaterials as prospective diagnostic tools, therapeutic remedies, or tissue substitutes based on chemistry-driven processes seen in nature. The majority of recent review studies have treated surface physico-chemical engineering and biological principles of biomaterial design as separate subjects, obviating the importance of surface chemistry in this field. In this review, we examined the biocompatibility in the context of surface proprieties, what it is and how to assess it. The paper contains the results of our published work but also the most representative results from other authors regarding the engineering concepts for developing biomaterials, with a focus on surface physico-chemistry (Figure 1). Finally, the existing tactics for increasing the chemical and physical interact.

## 2. Chemical Modification Techniques

### 2.1. Wet Chemical Techniques

At the microscale and nanoscale, wet chemical methods are helpful for better controlling the crystal phase and surface shape in a simple manner and allowing the insertion of biological and organic components to create a wide range of biological functions [2]. The surface enrichment with functional groups such as -NH_2_, -OH, and -COOH is a reliable technique for influencing cellular activity, and it can be accomplished through hydrolysis or aminolysis processes [3].

#### 2.1.1. Hydrolysis

**Figure 2:**
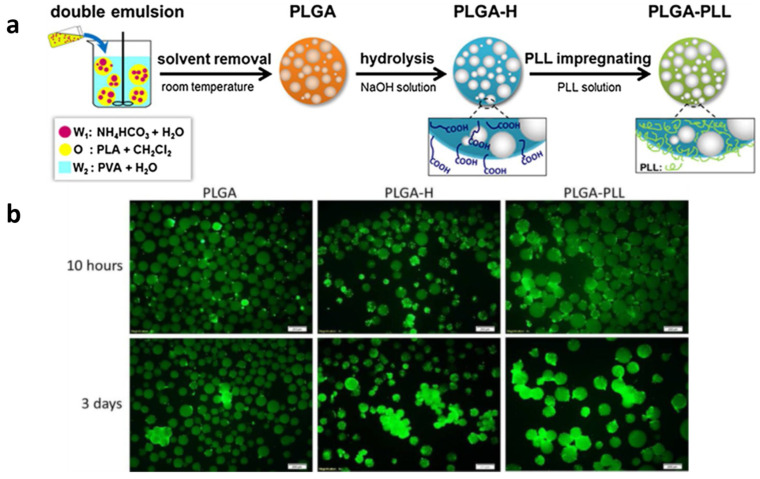
The PLL modified porous PLGA microspheres. (**a**) Schematic illustration of the formation process. (**b**) The microspheres after culturing with MG63 for different times; fluorescence micrographs scale bar 200 μm. Reproduced with permission from Ref. [4]. Copyright 2017 Elsevier B.V.

Yuan and coworkers prepared porous PLGA microspheres by a modified emulsion solvent evaporation method, and they subsequently functionalized the microspheres surface by hydrolysis (Figure 2). PLL was then utilized to modify porous PLGA microspheres using a simple dipping procedure to stimulate cell growth on them. Finally, modified PLGA microspheres were cultured in vitro to assess the impact of PLL coating on cytotoxicity, adhesion, and morphology [4] (Figure 2).

Alkaline hydrolysis in aqueous solution (0.01–0.05 M) permits the limiting of the hydrolysis to the surface of polymers [5,7,8]. A number of composite materials can also be surface-hydrolyzed, having been prepared from polylactic acid PLA [7,8,9], poly(ethylene terephthalate) PET [10], and poly(l-lactide-co-glycolide) PLGA [11]. The PLA film was prepared by standard solution casting technology and modified by the following surface alkali–acid hydrolysis method. First, the PLA film underwent a surface alkali hydrolysis by being immersed into a mixture of 10 g/L sodium hydroxide solution and absolute ethanol with the same volume for 1 h at room temperature. Then, the treated film was washed with 0.5% citric acid and deionized water [8].

PET films were immersed in NaOH (4 mol/L) at 70 °C under constant agitation at 750 rpm for 3 h and then washed in HCl (1 mol/L) until the pH was neutral. Hydrolysis is a simple way to obtain anionic—COO− groups onto a PET surface hydrophilicity; it is well known that it is strongly influenced by surface composition and morphology and subsequently by surface hydrolysis. It is a clear decrease in the water contact angle of the surface film as the hydrolysis time increases [9], as was expected, due to the increase in the hydrophilic carboxyl and hydroxyl groups on the hydrolyzed surface [12]. PET samples were hydrolyzed using 0.25 N sodium hydroxide in water/acetonitrile (1:1) solution at 60 °C. After that, the hydroxyl groups were completely oxidized by potassium permanganate (5 g in 100 mL 1.2 N sulfuric acid) at 60 °C. Then, the carboxylated PET films were washed with 4 N hydrochloric acid. Deionized water was used to wash the carboxylated PET thoroughly to remove any residual acids on the surface [13].

Modified electrospun PLGA of the scaffolds was carried out using EDC/NHS. Briefly, the fiber surface was hydrolyzed by immersing freeze-dried fibers in 0.01 N NaOH solution for 30 min at 25 °C. After surface hydrolysis, scaffolds were washed with DI water three times to remove additional NaOH. A solution containing EDC (40 mM) and NHS (80 mM) in buffer (pH = 6) was added to the hydrolyzed scaffolds and kept at 4 °C for 4 h [11].

Baba et al. [14] modified the surface film of the PLA: first, the PLA films were immersed in sodium hydroxide (NaOH), and after washing with distilled water and ethanol/water solution, by introducing amino functional groups, were transferred into a mixture of EDC/NHS solution. Then, the films were washed in distilled water and finally immersed in other poly(ethylene imine) solution at pH 7.4 [14].

#### 2.1.2. Aminolysis

The aminolysis technique is commonly used with alkylamine [15,16] in order to introduce primary or secondary -NH_2_ groups onto the surface of polymers [14,15,17,18,19].

Drobota and coworkers performed the aminolysis functionalization technique to activate the surface of PET films using alkylamine (triethylenetetramine (TETA) and tetraethylenepentamine (TEPA)), creating new binding sites in the ”sandwich model”. This functionalization is in one step. Then, after physically removing alkylamine adsorbed by ethanol, the immobilized collagen allows for the formation of a crosslinking surface, the cell adhesion and migration on biomaterial surfaces depending on the previous treatment [15].

**Figure 3:**
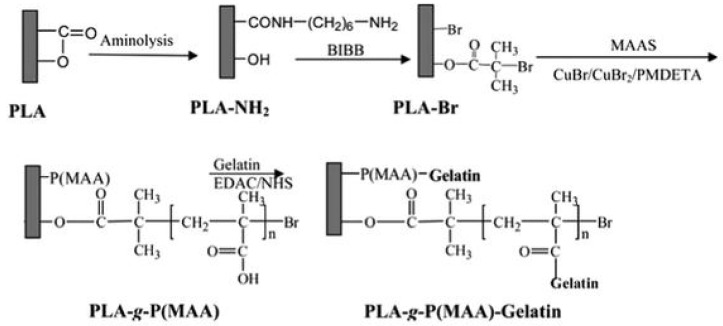
Illustration of the PLA aminolysis surface reaction. Reproduced with permission from Ref. [19]. Copyright 2013 The Royal Society of Chemistry.

Figure 3 presents the reaction of free amine and hydroxyl groups on the aminolyzed PLA film surface to obtain the PLA-Br surface via ATRP of sodium (meth)acrylate (MAAS) on the PLA-g-P(MAA) surface and the PLA-g-P(MAA)-gelatin surface [20].

Surface aminolysis of polyethersulfone (PES) membranes was performed via immersion in an aminolysis solution (10.0 wt % diethylenetriamine (DETA) in water). Then, after removing PES-NH_2_ membranes physically adsorbed by ethanol, they were incubated in a carboxymethyl cellulose (CMC) or SCMC (solution containing EDC/NHS and morpholine ethanesulfonic acid as solvent) [21].

Poly-L-lactide (PLA) nanofibers were modified, and nanofibrous 1,6-hexanediamine/isopropanol solution was aminolyzed at 50 °C at for 1, 3, 5, 7, 10, and 15 min. After aminolysis, the aligned PLLA nanofibrous scaffolds were rinsed with MilliQ water for 4 h at room temperature to remove free 1,6-hexanediamine [7,20].

Nashchekina et al. used polycaprolactone PCL films with a thickness of 5 µm, and they functionalized the surface of PCL with arginine. Due to the poor wetting of the hydrophobic PCL films, which makes it difficult for arginine molecules to approach the PCL molecules, the reaction was very sluggish in water, the most polar solvent in this study. The reaction rates were substantially higher in the less polar alcohols, with isopropanol achieving the greatest rate. It has previously been demonstrated that the pace of aminolysis reaction rises with decreasing solvent polarity, and that in the presence of methanol, the highest number of amino groups is immobilized on the surface of PCL films [22]. Jeznach et al. used three types of polymers for fabrication of nanofibers and films: poly(caprolactone) (PCL), poly(l-lactide) (PLLA), and poly(l-lactide-co-caprolactone) (PLCL) 70:30 solutions in HFIP. Samples were immersed in a 6 percent *w*/*v* solution of 1,2-ethandiamine (ED) in isopropanol for 5 and 15 min at 30 °C (sample/diamine solution ratio: 1.5 mg/mL). The efficiency of aminolysis for several aliphatic polyesters (PCL, PLCL, and PLLA) in the form of nanofibers and films under the same processing circumstances was found. The ranking of aminolysis efficiency from highest to lowest depended on the type of polymer: PLLA, PLCL, and PCL. Different ratios of ester to alkyl groups, primary crystallinity, and NH_2_ recombination rate are all part of our explanation for this sequence of polymer susceptibility to aminolysis [23].

### 2.2. Layer-by-Layer

**Figure 4:**
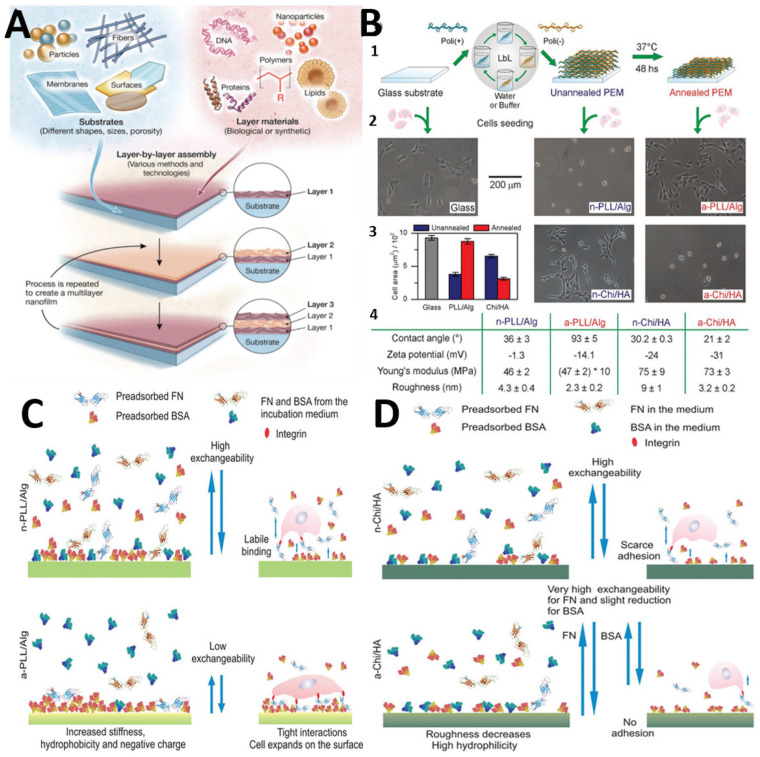
Versatility of layer-by-layer assembly. (**A**) Schematic illustration of LbL assembly. Reproduced with permission from Ref. [24]. Copyright 2015 The American Association for the Advancement of Science. (**B**) Scheme of the assembly and annealing protocol, phase contrast images of C2C12 cells adhered on different substrates, and average cell adhesion spreading area from cells seeded on different substrates and physicochemical properties of PEMs. (**C**) Scheme of protein adhesion mechanism and the effects on cell adhesion characteristics for nPLL/Alg and a-PLL/Alg. (**D**) Scheme of protein and cell interactions with n-Chi/HA and a--Chi/HA PEMs. Reproduced with permission from Ref. [24]. Copyright 2015 The American Association for the Advancement of Science.

The capacity to adjust film thickness by nanometers and the wide range of materials available for covering planar and particulate substrates have piqued interest in layer-by-layer (LbL) thin film assembly [24] (Figure 4A). Electrostatic attraction was used to alternately deposit positively and negatively charged polymers on the surface of a solid substrate, resulting in thin films. LbL assembly is a simple, effective, reproducible, flexible, and highly versatile method for modifying surfaces and fabricating robust and highly ordered nanostructured functional polymeric thin films and nanocomposites over any type of substrate with multilayered architecture containing the arrays [25]. The construction of electrostatically assembled LbL films incorporates dendrimers and different types of polymeric materials. Layer-by-layer (LbL) multilayer assembly is able to provide surface coating on precisely controlled scales (from a few nanometers to several micrometers) [26]. In the LbL technique, oppositely charged polymers (polyanions and polycations) are deposited on the charged substrates via an electrostatic force [27].

Because dendrimers frequently contain charged surface groups such as amine and carboxyl residues, it is possible to construct (a) dendrimer/polymer and (b) dendrimer/dendrimer LbL films through electrostatic bonding. Hybrid LbL assemblies containing dendritic macromolecules (dendrimers and dendrons) have been explored. LDH nanosheets (layered double hydroxides) offer a large amount of potential for integrating negatively charged organic molecules into limited arrays. The exposure of substrate to solutions containing oppositely charged species results in their electrostatic deposition and formation of ultrathin and uniform films [28].

Different types of polymeric materials, such as azobenzene polymer [29], poly(acrylic acid) (PAA) [30], poly(styrene sulfonate) (PSS) [31], sulfonated poly(aniline) [32], sulfonated poly(aniline), and poly(glycerol) [33] have been used to make LbL films containing dendrimer and synthetic polymers (glycerol).

A good example is Kim and Bruening, who used poly(amidoamine)/poly(acrylic acid) PAMAM/PAA films. They used PAMAM solution at pH 8 and PAA solution at pH 4, where both PAMAM and PAA were only partially charged, to obtain the highest film thickness. The films’ bilayer thickness was deposited at different pH levels. Electrostatic interactions with polyanions were used to incorporate PAMAM and poly(propylenimine) (PPI) dendrimers with positive surface charges into LbL structures [34].

Muzzio et al. used the LbL technique to obtain a multilayer film by preparing a solution from poly-l-lysine (PLL) and sodium alginate (Alg), and another one from chitosan (Chi) and hyaluronic acid (HA) (Figure 4B). All polyelectrolyte solutions were prepared at a concentration of 1 mg/mL with the exception of PLL, which was prepared at 0.01 mg/mL. In order to maximize opposite charge interactions, a reorganization of the polyelectrolyte chains was induced by the annealing process.

Figure 4 illustrates the changes in cell adhesion and in the physicochemical properties of PEMs induced by thermal annealing. Proteins have a stronger interaction with PEM surfaces on annealed PLL/Alg PEMs; the exchangeability is diminished; and FN, alone or in combination with BSA, has a stronger interaction with PEM surfaces. The exchangeability experiments on annealed Chi/HA PEMs show an increase in the amount and strength of BSA adsorption, but a negative effect on FN adsorption. When compared to n-Chi/HA, a-Chi/HA has lower cell adhesion properties. Chi/HA PEMs, unlike PLL/Alg-based PEMs, do not appear to facilitate BSA and FN collaboration in the adsorption process. Changes in the topographic features of deposited proteins on annealed PLL/Alg suggest that the proteins’ 3D structure has changed [35].

Schlenoff and co-workers suggested differences in cell adhesion between poly(diallyldimethylammonium chloride)/poly(sodium 4-styrenesulfonate) (PDADMAC/PSS). PDADMAC/PSS indicates 20 alternating layers of PDADMA and PSS, starting with PDADMA, using 1.0 M NaCl in both polyelectrolyte solutions. The dipping time in each polyelectrolyte solution was 5 min, followed by three consecutive water rinses for 1 min, after which the polyelectrolyte multilayer was allowed to dry in a clean room. The stability of adsorbed proteins is linked to multilayers of varied thickness and surface charge concentrations. Cell adhesion is connected with the lability of deposited BSA rather than the amount of BSA deposited, according to scientists [36].

**Figure 5:**
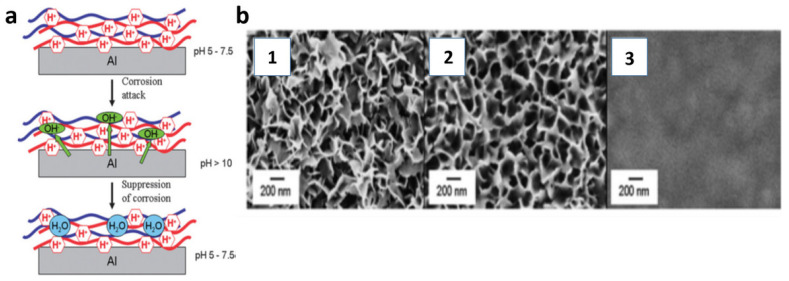
(**a**) Illustration of mechanism of buffering activity of polyelectrolyte coating. (**b**) The SEM images of the aluminum surface showing that when the number of polyelectrolyte multilayers increases, a homogeneous polyelectrolyte coating form. Reproduced with permission from Ref. [37]. Copyright 2008 The Royal Society of Chemistry.

Andreeva et al. deposited oppositely charged (PEI/PSS) polyelectrolyte (PE) multilayers on aluminum surfaces [37] (Figure 5). The corrosion processes on the aluminum surfaces were blocked due to the pH-buffering ability of polyelectrolyte-based LbL coatings. They presented a method of corrosion protection based on the buffering activity of a combination of a strong negative polyelectrolyte, poly(styrene sulfonate) (PSS), and a weak positive polyelectrolyte, poly(ethyleneimine) (PEI), deposited on aluminum alloy in a layer by-layer (LbL) manner consisting of weak polyacids or polybases that can be a prospective buffering system for metal protection. Even the deposition of two (PEI/PSS) bilayers (Figure 5(b2)) produces a protective covering that closely mimics the surface morphology of the original aluminum alloy (Figure 5(b1)), resulting in a very thin working layer. By increasing the number of deposited layers to 10 and filling the pores on the aluminum surface with polyelectrolyte multilayers, the surface roughness is minimized. The (PEI/PSS)10 coating (Figure 5(b3)) provides a dense, uniform, and defectless protective coating that can provide the buffering and barrier protection properties that polyelectrolyte multilayers are known for. It is critical to remember that the metal surface must be pre-treated first. PSS is a strong negative polyelectrolyte, having good adhesion properties to the metal surface without pronounced buffering ability, while weak positive PEI contains neutral and charged amine.

### 2.3. Surface Graft Polymerization

Surface graft polymerization is an efficient approach to modify surface of materials by introducing multifunctional groups and providing long-term chemical stability owing to possibility of covalent immobilization of bioactive molecules on the surface [38].

The modification of substrate surfaces is essential for numerous applications. A polymer brush is an assembly of polymer chains that are tethered at one end to a surface or an interface. The grafting of polymer on a substrate surface provides a practical tool for surface modification and functionalization [39].

**Figure 6:**
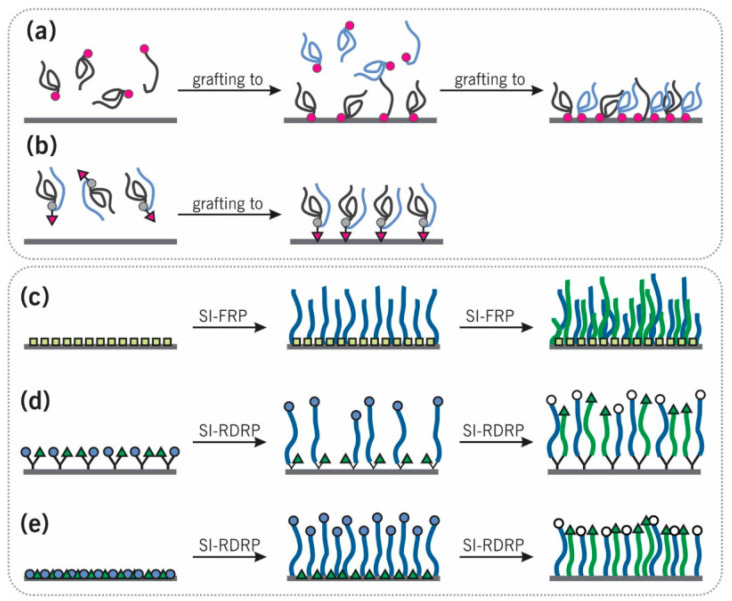
Illustration of different grafting synthetic routes. (**a**) Step-wise “grafting-to” of individual homopolymers, (**b**) “grafting-to” of Y-shaped diblock copolymers, (**c**) step-wise “grafting-from” via surface-initiated free-radical polymerization (SI-FRP) using non-selective initiators, (**d**) “grafting-from” via surface-initiated reversible-deactivation radical polymerization (SI-RDRP) using two disparate co-deposited initiators, and (**e**) “grafting-from” via SI-RDRP using Y-shaped bifunctional initiators. Reproduced with permission from Ref. [41]. Copyright 2020 Li and Pester under CC BY 4.0.

Chemical surface modification methods based on grafting can be categorized into “grafting-to”, “grafting-from” and “grafting-through” approaches, commonly used for the covalent grafting of polymers [40,41] (Figure 6a). This approach can be used to prepare polymers that have well-defined molecular weights and molecular weight distributions and can be grafted onto surfaces. In the grafting-to approach, end-group-transformed polymer chains react with the functional groups of substrates and form grafted polymer chains. It has been established that conventional and controlled radical, anionic, and other polymerization or copolymerization techniques are “tried and true” methods.

The “grafting-to” approach is very common in the application of polymeric biomaterials, providing advantages over other techniques including the ability to characterize the structure of the grafting polymers. “Grafting-to” [41] can be obtained by (i) two individual types of chain sequential or simultaneous co-deposition (see Figure 6a) or (ii) a “Y-shaped” diblock copolymer deposition with a functional group located among the two disparate polymer segments (see Figure 6b).

By co-depositing two pre-synthesized polymers at the same time, the “grafting to” approach can be used. Poly(ethylene oxide) (PEO)/poly(acrylic acid) (PAA) brushes, for example, were made by combining a gold substrate with a combination of thiol-terminated PEO and PAA instead of sequential depositing [42]. PEO and PAA chains were grafted at the same time, and the mass ratio in the mixture was used to adjust the fraction of each component. The polymer solution for grafting (PEO, PAA, PEO/PAA 10/90, or PEO/PAA 50/50) with stock solutions of polymers were prepared at a concentration of 5 g/L in ultrapure water and were diluted in water to the desired concentration of 1 g/L prior to each experiment. The role of the amount of PEO in the polymer is regulated by changing its molar mass or its fraction in the solution used for polymer grafting. The adsorbed proteins can be completely removed only from the PEO/PAA mixed brushes with a minimum of 25 PEO units per nm^2^. The desorption is believed to be triggered by the exposure of PEO chains upon PAA shrinking [42].

Nebhani et al., using this “grafting-to” method for the surface modification of divinylbenzene (DVB)-based microspheres, quantified the grafting densities achieved via a combination of reversible addition–fragmentation chain transfer (RAFT) polymerization and rapid hetero-Diels–Alder (HDA) chemistry [43].

For example, polymer can be grafted onto different types of polymeric substrates to modify the biomaterial. Many polymers have been used to generate new biomaterials such as polyethylene terephthalate (PET) [44,45], polyethylene (PE) [46], polypropylene (PP) [47], polyurethane (PU) [48], polyvinylchloride (PVC) [49], polyvinylidene fluoride (PVDF) [50], poly(vinylmethyl siloxane) (PVMS) [51], and polydimethylsiloxane (PDMS) [52].

These studies revealed that the grafting-to method could also promoting a high-graft-density grafted layer.

Hayder et al. [45] used the coupling agent N,N′-dicyclohexylcarbodiimide (DCC) for obtaining two separate layers covalently immobilized of chitosan and dermatan sulfate (DS)—an anionic polysaccharide—on PET. PET surfaces were hydrolyzed using a mixture of 0.5 M NaOH and acetonitrile (1:1, *v*/*v*) at 60 °C, then oxidized by KMnO_4_ (50 g/L) in 0.6 M H_2_SO_4_. Functionalized PET surfaces (PET–COOH) were immersed in 20 mL of acetonitrile solution and sonicated for 15 min. The activation of the carboxylic groups formed on the surface was performed by the immersion of the samples in a solution of DCC in acetonitrile (10 mg/mL) at room temperature. To remove excess reactant, samples were rinsed in DS solution (1 mg/mL) by dissolving dermatan sulfate powder in PBS at pH 7.4. Chitosan was first dissolved in 1% (*v*/*v*) aqueous acetic acid (pH 5.0) before increasing the pH up to 7.4. The volume of the buffered solution was adjusted with PBS to obtain a concentration of chitosan of 1 mg/mL. PET disks with activated surfaces were treated after the first layer immobilization, which was either DS or chitosan, and PET–DS and PET–Chito samples were activated one more time using the same coupling agent (DCC) and the same method.

Briefly, PET–DS and PET–chitosan samples were immersed in a solution of DCC in acetonitrile (10 mg/mL) then washed in acetonitrile solution. Activated samples were immersed either in chitosan solution or DS solution, as previously described, to immobilize the second layer. After washing, PET co-grafted by DS and chitosan were obtained and named, respectively, PET–DS–Chito and PET–Chito–DS. For PET–Chito and PET–Chito–DS, they presented contact angles lower than native PET, and the immobilization of the second layer further increased the surface wettability due to the presence of polar functional groups. The comparison between the wettability of new final materials obtained showed that PET–DS–Chito is more hydrophilic than PET–Chito–DS [45].

In the grafting-from approach, polymer chains propagate from surface-attached initiators. The “grafting-from” method means using an active site on the substrate surface to initiate the in situ polymerization reaction of the monomer. The “grafting-from” method describes the growth of polymer chains from a substrate surface using surface-attached or self-assembled initiator moieties, which is sometimes called “surface-initiated polymerization” [53].

First, initiator-bearing self-assembled monolayers are immobilized onto substrate surfaces. Notably, recent reports have suggested that these monolayers can be immobilized on almost any surface with a suitable anchor functionality. Then, the initiator-bearing surfaces, catalysts, and monomers are mixed together to graft polymer chains. Generally, the conformations of the polymer chains grafted onto a substrate surface can be divided into four categories: “pancake”, “mushroom”, “brush” and “highly stretched brush” [54].

Arslan and Günay [55] obtained modified poly(ethylene terephthalate) PET fibers using the grafting-from approach. PET fibers were graft copolymerized using 4-vinylpyridine (4-VP), methacrylic acid (MAA), and glycidyl methacrylate (GMA). Then, over pyridine, HCl was bound to fibers. They were chloride treated after MAA was aminated. Furthermore, triclosan, a powerful chemical agent, was added to MAA and GMA. A total of 100 mL polymerization tubes with nitrogen gas inlet were utilized for the graft copolymerization of vinyl monomers on PET fibers. The addition of 2 mL benzoyl peroxide (Bz_2_O_2_) solution in acetone at an adequate concentration yielded a total volume of 20 mL, and graft polymerization was carried out under a nitrogen environment in a condenser. For the grafted fibers, a 30 mL combination of 20% hydrogen peroxide and acetic acid was used. 4-VP-g-PET fibers were modified by oxidation and chlorination, and the obtained mixture was spun at 25 °C in a water bath.

Amination and chlorination of aminated fibers and binding of triclosan to the PET fibers grafted with MMA were all used to modify the MMA-g-PET fibers. Hexamethylene diamine (HMDA) and tetraethylene pentamine (TEPA) were utilized as amines in the amination process. In a 50 mL mixture of 50% HMDA–ethanol and 50% TEPA-2-propanol, grafted fibers were applied at 30 mL. This combination was spun in a water bath at 30 °C. Aminated fibers were washed in methanol and water before being dried at 50 °C. Aminated fibers were soaked in a 30 mL combination of 25% hydrochloric acid and water for chlorination. This combination was spun in an incubator with a shaker at 25 °C. Chlorinated fibers were washed in water and dried at 50 °C in an incubator. The fibers were modified with triclosan to make GMA-g-PET fibers. AA showed antibacterial action in both solid and liquid culture experiments. When aminated fibers were quarternized with chlorine, the activity of the fibers was boosted and the zone diameter rose. The triclosan-modified fibers exhibited the largest zone diameter. The results of the disc diffusion sensitivity test were validated by those of the liquid culture test, and the results were quantified. According to the growth curve of *S. aureus* inoculated with 0.1 g of Trc-MMA-g-PET fibers with added liquid media, the polymer has an antibacterial action.

Mabry et al. reported [56] the obtaining of surfaces containing using poly(ethylene glycol) (PEG) were prepared by grafting α-methoxy-ω-triethoxy PEG silane to fused silica wafers. The varied grafting density onto PEG was adjusted during functionalization. After washing, the wafers were functionalized with α-methoxy-ω-triethoxy PEG (1 mM) in 100% acetone, 75% acetone/25% diethyl ether, or 50% acetone/50% diethyl ether [57]. They observed that when the grafting density increased, the adsorption rate of protein decreased constantly further with the rate at the highest grafting density on the surface [56].

Some different types of controlled polymerization methods utilized for the “grafting-from” on surface are using surface-initiated atom transfer radical polymerization (SI-ATRP) [58,59,60], reversible addition–fragmentation chain transfer polymerization (RAFT polymerization) [61], and nitroxide-mediated polymerization (NMP) [58].

The surface-initiated atom transfer radical polymerization (SI-ATRP) grafting-from method arises from its ability to precisely control the structure and properties of the prepared hybrid material. For example, methyl methacrylate (MMA), regarded as one of the most promising monomers for the formation of polymer brushes via SI-ATRP, has been widely investigated [20].

RAFT polymerization differs in that it can operate with various monomers in various reaction conditions, owing to its controlled molecular weight polymers with very narrow polydispersities. The PDMSe substrates have been suitably modified, enabling additional applications in the investigation of the biofouling responses of marine organisms to chemically functionalized surfaces [62].

Photoinduced electron/energy transfer-RAFT (PET-RAFT) technique, for initiating polymerization using visible light that combines green chemistry and the level of control provided by RAFT in manufacturing polymers for surface modification, was employed for green and precision polymer manufacturing. In a recent study, Zhou et al. used [2-(methacryloyloxy) ethyl]dimethyl-(3-sulfopropyl) ammonium hydroxide] (MEDSAH) as a kind of sulfobetaine monomer to construct an antifouling surface on a PVA hydrogel via PET-RAFT polymerization [63].

Nitroxide-mediated polymerization (NMP) is another living polymerization technique based on the reversible capping of the active chain-end radical with a nitroxide leaving group [58]. NMP was used to graft (functionalized) polystyrene from bulk or surface-functionalized polyolefins, according to Domenichelli et al. Macroinitiators included high-density polyethylene and a poly(ethylene-co-olefin) copolymer (EOC) modified with various functional 2,2,6,6-tetramethylpiperidine-1-oxyl (TEMPO) radical derivatives. These findings suggest that functional polyolefin-alkoxyamines can be thought of as “active macromolecules” that can be used as macroinitiators to make graft polymers with certain structural properties. Further monomers (such as methacrylates, dienes, and halogenated alkenes) may also be attempted to prepare novel graft copolymers [64].

In the grafting-through process, polymer chains propagate from surface-attached double bonds. The “grafting-through” approach is used frequently on the basis of surface-attached monomer groups, the surfaces featuring a self-assembled monolayer containing polymerizable groups.

In this process, polymer chains grow in solution in the beginning and then propagate, while surface-bound monomer units are inserted into the growing chains. When free or surface-attached units are introduced into the growing chains, the process could be called a “grafting-through reaction”.

Henze and coworkers studied the variations in the temperature, initiator, or monomer concentration in solution, polymerization time, and surface concentration of the surface-attached monomer that influences the formation of the surface-attached polymer layer in terms of this “grafting-through” reaction [53].

## 3. Physical Modification Techniques

### 3.1. Plasmas Techniques

A plasma is a partly ionized gas containing free electrons, ions, radicals, and neutral particles (atoms and molecules). Some of these particles may be in an excited state; they can return to their ground state by photon emission, which produces typical plasma light emission. Plasmas are typically obtained when gases are excited into energetic states by radio-frequency, microwaves, or electrons from a hot filament discharge [65]. However, the plasmas predominantly used for surface treatment are cold non-equilibrium plasmas where the temperature of the electrons (related to their translational energy, 1–10 eV) is higher than that of ions and molecules or radicals, and where only a few percent of the molecules are ionized. The energy of most electrons is in the range of 1–5 eV, similar to that required to break simple organic chemical bonds.

Plasmas are certainly the most widely used method for surface modification of polymers. Corona discharge, a related method, involves the ionization of a gas surrounding a conductor maintained at high potential generating cold plasma (industrial applications are similar to those of plasmas). When a surface is in contact with a plasma, two types of modifications are possible: (a) simple gases (Ar, H_2_O, O_2_, N_2_, etc.) produce reactive species that react with the polymer surface; for example, oxygen plasma permits the hydroxylation of the surface of polymers, and (b) if organic molecules (such as saturated or unsaturated hydrocarbons) are used to generate a plasma, polymeric films are grown from the surface [65].

Although many studies are concerned with plasma for polymer surface modifications, many of them involve radio-frequency (RF) plasma [66,67], with only a few using direct current (DC) plasma [68]. A DC plasma is more stable in comparison with RF/MW/inductively coupled plasmas, with it being easy to control and to diagnose a DC plasma by comparing it with RF/MW/inductively coupled plasma. Moreover, a pulsed power supply is technically more complex than a DC source and compromises the reproducibility of the process. The main disadvantages of DC plasma systems are: (1) the contamination of the discharge products by the filament, and (2) the variation of the discharge characteristics with the geometry of the discharge tube [69].

Polymers such as polyethylene (PE), polypropylene (PP), poly(ethylene terephthalate) (PET), polylactic acid (PLA), poly-ε-caprolactone (PCL), poly(lactic acid-coglycolic acid) (PLGA), and poly(hydroxybutyrate) are materials with good bulk properties for biomedical applications [65].

PE both sides of 5 × 5 × 0.1 cm PE foils were exposed to nonthermal non-equilibrium air plasma by using the following plasma reactors: Femto (Diener electronic, Ebhausen, Germany) operating at a frequency of 40 kHz, with a chamber of Ø 150 mm, 320 mm length, operated at a frequency of 2.46 GHz. Pressure, power input, and carrier gas feed rate were the same in every experiment, viz., 40 Pa, 50 W, and 20 sccm (standard cubic centimeter per minute), respectively. The duration of treatments was 1 and 2 min. For instance, the contact angle of deionized water decreased at least 26 [^o^], and the most drastic change was on 61 [^o^], which indeed denoted an improvement of surface wettability [70]. In PE hollow fiber membrane modified using Ar gas, controlled at 0.4 mL (STP)/min, with a pressure in the system (between 10 and 60 Pa), plasma generated power (between 20 and 80 W) and different time (between 30 and 300 s) [71]. The contact angles for outside membrane surfaces were close to 60 [^o^], far less than that of the virgin membrane 120 [^o^] [71].

Bitar et al. [72] modified PP foils with a thickness of 50 μm from Goodfellow (UK), used without any pre-treatment. By selecting this motion speed of the needle head, every single treatment took approximately 0.4 s. As a result, the typical treatment time of each line segment of 1 mm was approximately 20 ms. By changing the amplitude of the applied high voltage, the discharge power can be varied between 0.12 W (for 3.4 kV) and 0.81 W (for 5.2 kV). Using an air driven discharge induces sub-millimeter changes to the surface properties of PP substrates by modifying a pre-defined area. After applying a range of powers (0.12–0.81 W), the results showed that the WCA progressively decreased PP film with increasing power from 101.5 [°] ± 1.7 [°] for the untreated ample to 52.8 [°] ± 6.0 [°] for an applied power of 0.81 W. Surface saturation regions were found, but they differed depending on the applied plasma parameters, which could be linked back to combined changes in surface chemistry and roughness. Both XPS and AFM measurements confirmed these changes; depending on the applied power and number of treatment repeats, PP surface topography could be modified to different extents. Above the threshold value of 0.14 W, a granular morphology was found that was more pronounced. Increasing power and, to a lesser extent, increasing treatment repeats will lead to fewer, larger granules. The incorporation of oxygen groups into the modified line on the PP surfaces was visualized by XPS analysis. The highest applied power (0.81 W) and the highest number of treatment distribution of oxygen was found after analysis of horizontal cross-sections of the modified plasma. Increasing power and treatment repeats resulted in higher percentages of incorporated oxygen [72].

While PE and PP that are devoid of functional groups were treated with a water plasma to give hydrophilic surfaces, the hydroxyl groups formed on the surface were detected by the decreased water contact angle from 97 [^o^] to 54 [^o^] and by the appearance of an O1s XPS peak (C–OH/–C = 13%) [65].

**Figure 7:**
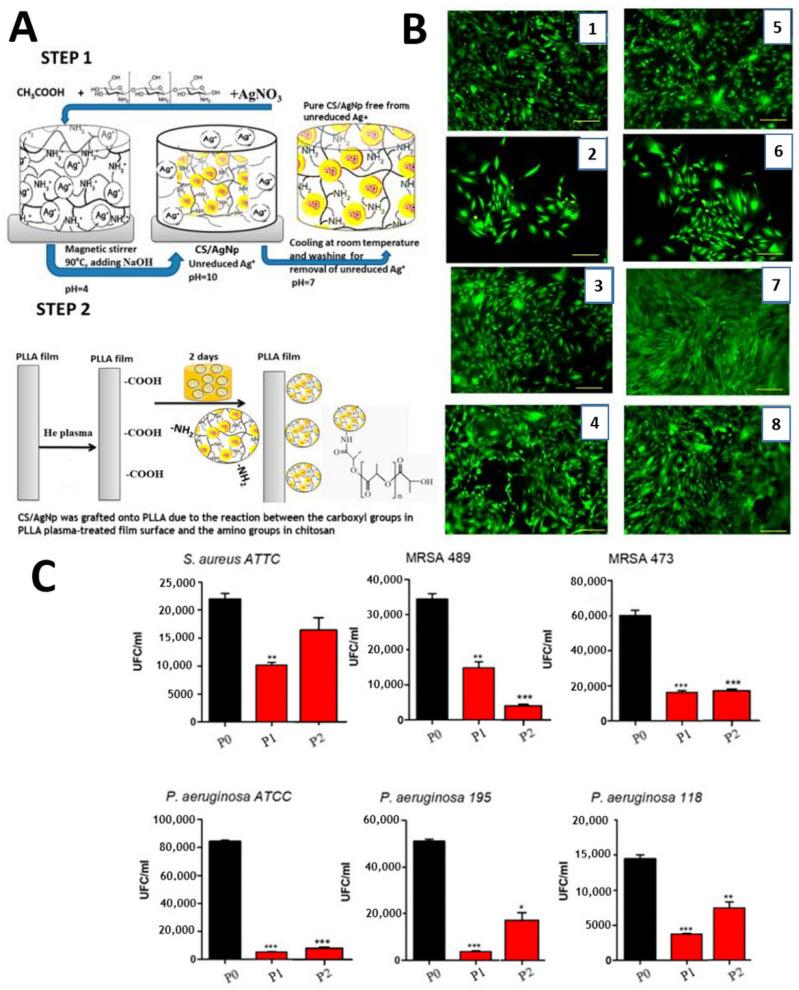
(**A**) Schematic representation of the two-step treatment. (**B**) Cell culture (cell line MC3T3-E1) after 48 h. (**C**) Strain adherence of *S. aureus* and *P. aeruginosa* (ATCC, clinical isolates) to polylactic acid films, *, ** and *** represents the magnitude of antimicrobial activity. Reproduced with permission from Ref. [73]. Copyright 2021 Aflori under CC BY 4.0.

Films with increased antibacterial capabilities were created using a simple two-step approach that included plasma treatment and then wet chemical treatment of poly-L-lactic acid (PLLA) films in a solution containing chitosan-based silver nanoparticles (Cs/AgNp) (due to the presence of chitosan nanoparticles with silver on their surface) (Figure 7). Aflori and coworkers [73] treated 100 µm thickness, 2 cm × 1 cm polyethylene terephthalate (PET) using gas helium at a pressure of *p* = 10^−2^ mbar. Plasma density had a value of n = 108–109 cm^−3^ and was measured with a plane Langmuir probe. From the current–voltage characteristic of the same probe, the researchers found two groups of electrons with the energies of about 2 eV and 0.2 eV, respectively. Under the plasma action, the chains scindation will appear on the PET surface, generating polar groups such as COO, OCO, and OH. The elemental composition using XPS measurements and the treated and untreated PET film presented an O/C ratio equal to 0.19, while after the plasma treatment, plasma increased to 0.25 [69].

The surface wettability of PET films treated with radio frequency (RF) atmospheric-pressure plasma, which combined plasma activation and hexamethyldisiloxane (HMDSO)-based plasma polymerization, was achieved, resulting in stable PET surface properties ranging from highly hydrophobic to superhydrophilic. With Ar plasma activation and plasma polymerization in a single plasma source without configuration modification, PET surface wetting can be controlled from high hydrophobicity (greater than 140 [°]) to superhydrophilicity (<10 [°]). Moreover, the highly hydrophobic and superhydrophilic surfaces showed very good time-lasting stability with a negligible aging effect, and the HMDSO coatings were stable in aqueous media. The rough surface with Ra larger than 30 nm should also play a key role for these highly hydrophobic properties. The oxidation of the high concentration of CH_3_ groups on the surface caused by plasma activation should contribute to the superhydrophilicity for plasma activation as post-treatment of the plasma polymerization process [74].

CUTE-1MPR (Femto Science Inc., Gyeonggi, Korea) running at 40 kHz was used to cure AC discharge plasma. Before the working gas, synthetic air was injected at a flow rate of 20 sccm, and the chamber (140 × 200 × 110 mm; electrode area of 200 cm^2^) was evacuated to a base pressure of 20 mTorr. The glow discharge was then triggered at 50 W for 10–60 s. A laboratory-made setup consisting of two 18 cm diameter electrodes (electrode area of 254 cm^2^) with a 5 cm spacing was used to perform DC discharge (50 mA) plasma therapy. To ensure a steady ignition and sample surface treatment during DC discharge plasma modification, PET films were 1.5 × 2.5 cm (i.e., 3.75 cm^2^). Commercially available PET film samples (40 m, Turkey) were used. Wetability contact angle measurements were taken since plasma treatment is commonly used to promote surface hydrophilicity. Water drop wettability contact angle ranged from 80 [°] (non-treated PET films) to 17 [°] for AC plasma or practically full water drop spreading (10–12 [°]) for DC discharge plasma treatment [75].

Luque-Agudo et al. [76] modified the surface of PLA films with plasma treatments, both of a reactive gas (oxygen) and of an inert gas (argon): power of 0–24 W, frequency of 100 kHz, treatment time of 10 min, and gas pressure of 0.5 bar. In terms of the WCA of the PLA untreated film, the WCA= 81 [^o^] ± 3 [^o^]: the WCA(O_2_) = 57 [^o^] ± 2 [^o^], which decreased by almost 30% (oxygen plasma), and WCA (A_r_) = 51 [^o^] ± 3 [^o^] by almost 37% (argon plasma), wherein the PLA surface became hydrophilic when exposed to plasma. That increase occurred in a nanometric scale, reaching surface roughness values (Rrms) of about 56.8 nm, which was remarkably higher than the surface roughness of the untreated material with Rrms 12.1 nm. The chemical modifications were different, and similar physical changes were observed for both plasmas: increase in surface roughness and decrease in hydrophobicity. However, the latter was more durable in the case of argon plasma, due to the lower mobility of the chains [76].

PCL films were treated at high RF power (30 W to the RF coil) for 60 min with oxygen plasma for a period of time of 30 min in order to compare oxidation levels; stabilized pressure was fixed at 250 mTorr (0.33 mbar). PCL films oxygen plasma treatment of the PCL led to changes in chemical structure in both surface morphology and roughness (RMS). The plasma treatment of the PCL film decreased the contact angle value from 75.7 [°] for PCL to 56.6 [°] by the surface oxidation action [77].

Electrospun PLLA nanofibrous scaffolds were treated for 60 s using PDC-002 plasma (USA) in the presence of oxygen. The chamber was evacuated to less than 10 mTorr before it was filled with gas, followed by the generation of glow-discharged plasma for a predetermined time [78].

Plasma treatment induced significant change on the water contact angle of nanofibers. The water contact angle of PLLA NFS was 128.2 [^o^] ± 2.3 [°]. It shows that PLLA is a hydrophobic polymer. After plasma treatment, it decreased to 48.5 [^o^] ± 3.3 [°]. This indicated that the surface of plasma-treated PLLA NFS became more hydrophilic. The introduction of oxygen-containing polar groups on the surface of nanofibers resulted in the significant change of hydrophilicity, which has been confirmed by multiple studies [79].

The literature on gaseous plasma activation of polytetrafluoroethylene (PTFE) is extensive, and gaseous plasma treatment of PTFE can result in increased or decreased hydrophilicity. Carbone et al. [65] used a microwave (MW) plasma jet in pure argon or in a mixture of argon (Ar) and oxygen (O_2_) at discharge power of 50 W and atmospheric pressure. After 15 s in argon plasma, the water contact angle (WCA) remained virtually unaltered; the WCA of the untreated PTFE sample was 110 [°]. The WCA was reduced to 90 [°] after a 600 s treatment, but when oxygen was given, the WCA did not decrease any more, but instead increased. Karoly et al. [80] reported a moderately hydrophilic character of PTFE after plasma treatments in air discharge at the frequency of 10–20 kHz with a 20 kV peak-to-peak voltage and a power of 320 W. The treatment times were between 30 and 600 s. The initial WCA, which was 108 [°], gradually decreased with increasing treatment duration, eventually dropping to around 65 [°] after 600 s.

Other researchers [81] used a discharge powered at a frequency of 25 kHz to treat PTFE films in different gases at times of up to 30 min. The original WCA (for untreated PTFE) was 80 [^o^]. The contact angle decreased with the increasing treatment time in oxygen for prolonged treatment, the lowest WCA was of 45 [°], followed by argon (49 [°]), nitrogen (51 [°]), and air (61 [°]) [81]. Plasmas with no oxygen introduced (at least not purposefully) or with a marginal oxygen content in the gas phase had the highest oxygen concentration in the polymer surface coating (well below 1 vol %). This finding is unique to the plasma activation of PTFE. The hydrophilic surface finish is commonly produced for most fluorine-free polymers by a brief treatment with a plasma sustained in pure oxygen or a combination of oxygen and a noble gas [81].

#### Adsorbtion Molecules

Chemical and physical surface modification of polymers can substantially enhance their biocompatibility due to their superior physicochemical properties, inhibiting bacterial growth with greater blood compatibility [82]. Plasma treatment can introduce polarized groups such as hydroxyl, carboxyl, amino, and sulfates on polymer surfaces using different reaction gases such as air, NH_3_, SO_2_, CO_2_, and other organic compounds [83]. Polymers in their pristine state are usually biologically inert, and thus some kind of a surface treatment is required to turn them into more advanced materials that induce a specific response in various biological molecules they come in contact with. Modulation of the surface properties of the polymer substrates, such as morphology and roughness, as well as physico-chemical composition, is necessary to achieve the desired interaction between the polymer and the biological agent [84].

By adjusting a number of plasma-processing parameters, such as discharge type, duration, and working gas type, it is possible to change the material’s surface characteristics, such as chemical structure, morphology, hydrophilicity, and surface charge [85]. It is well known that hydrophobicity increases protein adsorption to surfaces, but in some cases, it can also cause irreversible adsorption of proteins to the surfaces, while on the other hand, greater than normal hydrophilicity can prohibit protein adsorption, as well as plasma-induced biomolecule modification or damage on membrane molecules and intracellular molecules [86].

Surface etching depends on active plasma components affecting treated surfaces such as wettability, and the observed changes in surface chemical structure and morphology are sufficient to influence surface properties of polymers during a plasma treatment. The surfaces of polymers can be modified with simple functional (OH, NH_2_) groups, but there are many applications where more complex molecules must be attached to the surface; in these cases, a post-modification of the surface must be performed [65].

Demina et al. [75], after plasma treatment of PET films, were able to enhance the adsorption of chitosan (1% CH_3_COOH) from its solution. After incubation in chitosan solution, the films were thoroughly washed to remove any remaining “free” polysaccharide. Because of the presence of chitosan on the plasma-treated PET surfaces, these samples were fluorescently microscopically labeled with FITC, a fluorescein reactive to amino groups. The surface morphology of non-treated and plasma-treated films were not significantly altered as a result of chitosan immobilization [75].

On the surface of polymers for various biomedical applications, there are multiple variables that need to be controlled, such as shape, size, and its distribution. Aflori et al. immobilized collagen molecules on PET surfaces. After the He plasma treatment, the films were immersed in 3 mg/mL type I collagen/phosphate-buffered solution (PBS, pH 3.4) for 24 h at 24 °C. SEM images of the collagen buffer solution showed a surface divided into two zones, the first one being characterized by large structures from the buffer solution, while the collagen molecules aggregated forming dendritic structures. The collagen immobilization on plasma-treated PET was evidenced by heterogeneous surface coverage with large dendrites of collagen immobilized and small “grains” at short-time plasma-treated surfaces. At longer plasma-treated surfaces a relatively homogenous distribution of the collagen “grains” of different dimensions with a tendency to cluster was observed [69].

Wei and coworkers present the PP spun bond non-woven fabric with a good adsorption effect on copper ions, after the surface was activated by atmospheric pressure air plasma and then coated with chitosan solution. The water contact angle of untreated PP non-woven fabric was 136.6 [^o^] when treated with 120 W, which gradually reduced to 59 [^o^], indicating a transition from hydrophobic to hydrophilic state. Thus, the hydrophilicity was greatly improved, resulting from the generation of polar groups such as –OH and –COOH during the plasma processing under air atmosphere. The PP non-woven fabric showed complete wetting at the optimal plasma treatment technology at 180 W and 120 s. The surface morphology of the grafted fabric was much rougher, and small particles arising from chitosan and 1-vinylimidazole were attached to the surface compared with the pristine fabric, further indicating that the related functional compounds have been grafted on PP non-woven fabric. The surface morphology was much rougher, and small particles arising from chitosan and 1-vinylimidazole were attached to the surface compared with the pristine fabric, indicating that the related functional compounds have been grafted on PP non-woven fabric. The PP non-woven fabric prepared by plasma treatment and grafting modification had excellent hydrophilicity and copper ion adsorption performance, therefore having much potential application in the heavy metal wastewater treatment field [87].

The number of nanoparticles bound to the modified surface depends on the plasma and chemical pretreatment, the type of the material, and the grafted nanoparticles. Nanoparticles have different preferences when it comes to the chemistry and morphology of the surface, with higher concentration on pretreated substrates suggesting more binding for particles. The plasma pretreatment, as well as the final grafting of the noble metal nanoparticles, also leads to a significant change in zeta potential, due to the change in the surface charge of the polymer cause by the increase in polar groups in the surface layer. Pt and Pd nanoparticles caused a significant shift towards a positive charge, while Au nanoparticles, on the other hand, caused a dramatic decrease well below the values of the pristine polymer substrate, greatly increasing the conductivity of the grafted surface, which is also known to improve biocompatibility [84]. Drobota et al. [88] anchored the Au particles (in 0.1 mM PBS Au reactant free) after the ignition of Ar plasma (power 30 W, and the pressure of 10^−2^ mbar) at different treatment times of their low degradability rate and high active groups at the polymer surface. The tests were evaluated in comparison to the PET sample, and the results support the interaction of the components; the groups present on the surface; and the ions AuO-, AuNps, and O- groups, which increase the ability to form hydrogen bonds as determined by XPS measurements and biological activity. It can be explained by the structure of the films modified with Au nanoparticles: the gold is immobilized by physical boundaries only at the surface of the polymer in a thin layer, and we expect gold diffusion into the medium due to the presence of functionalities after plasma treatment [88].

### 3.2. Ultraviolet Technique

#### 3.2.1. Principle of the Technique

A light source that directs UV or visible light onto the prepared product is required for UV treatment. The substrates absorb UV energy from the light source, triggering a chemical reaction that swiftly transforms them into a solid functionalized surface, resulting in monomers and oligomers. Certain new properties are present on the surface of the polymer as a result of the reactive functional groups of the substrates that develop during UV light exposure. The final qualities are determined by the type of oligomer backbone, which might be epoxy, polyether, polyester, polyurethane, or other forms. Both ends of the oligomer molecules have functional groups that facilitate connection between molecules.

Light screeners, UV absorbers, excited-state quenchers, peroxide decomposers, and radical scavengers have been developed as stabilizing systems that rely on the action of stabilizers; of these, it is generally believed that excited-state quenchers, peroxide decomposers, and radical scavengers are the most effective. Polypropylene, polystyrene, polycarbonate, acrylonitrile styrene copolymer, and poly(methyl methacrylate) UV irradiation for varied periods have been studied by certain writers. The accumulated layer of the deteriorated species was created by photodegradation corresponding to the extinction coefficient in the UV region. UV irradiation is vital for controlling polymer breakdown. The radical process for the thermal oxidation of hydrocarbons is the basis for most recognized theories of polymer oxidation. The production of peroxy groups is involved in this process [89].

When the energy of UV light is equal or higher than the band gap energy of the sensing material, photogenerated electrons and holes can be produced by the absorption of UV light. In fact, the energy required to excite these electrons and holes is supplied by the light, instead of by heat [90].

The surface modification increases their application possibilities. In general, the polymers of modified surface have suitable surface wettability that affects other important properties (adhesion, biocompatibility, etc.) [91].

#### 3.2.2. Activation Effect

Silovská et al. [91] modified the surfaces of the polymer foils polyethylene terephthalate (PET), polypropylene (PP), and polyether ether ketone (PEEK) (50 µm thickness), which were activated by UV radiation, at room temperature (RT), using a UV lamp of the wavelength of 254 nm (UV-C, only UV). All samples were activated by UV for 10, 30, and 60 min and then were put into the methanol solution of cinnamaldehyde with different concentration (5, 10, and 20 wt %) outside for 60 min. Activation of polymer foil using UV-C radiation led to cleavage of the original polymer chains, and their oxidation created new reactive sites on the polymeric surface capable of the following grafting with cinnamaldehyde. Moreover, these steps alone play important role in wettability surface changes. In the case of PP (which consists of only from C and H atoms with no oxygen in original polymer chains), these steps lead to the increase in oxygen groups, the oxidation of the surface in comparison with the original PP molecule, and then to the decrease in the contact angle. On the other hand, the original chain of PET also consists of oxygen, and the subsequent grafting of the cinnamaldehyde molecule (C_6_H_5_CHCHCHO) contains less oxygen atoms and longer CH– chain in comparison with the original PET molecule that means the decrease in oxygen groups on the surface, as well as of the polarity and wettability, leading to the increase in contact angle. The contact angles of unmodified foils are (i) 75.3 [^o^] ± 1.3 [^o^] (PET) and (ii) 97.0 [^o^] ± 0.9 [^o^] (PP). The wettability of surfaces both of polymers activated by UV for 30 min and then grafted with cinnamaldehyde change the (i) 59.1 [^o^] ± 0.7 [^o^] (PET) and (ii) 104.7 [^o^] ± 1.2 [^o^] (PP). In the case of PET and PEEK trace amounts, some changes were induced on the sample surfaces by the UV treatment and also after grafting with cinnamaldehyde. The results were different: increase in oxygen concentration after UV treatment due to –OH group creation UV treatment showed the changes in surface properties of PET, PEEK, and PP foils after UV action and subsequent grafting with cinnamaldehyde; the changes were more significant after more than 30 min of UV modification. Both UV treatment and cinnamaldehyde grafting led to changes in surface chemistry, wettability, charge, and polarity. The analytical methods confirmed the successful UV activation and subsequent cinnamadehyde binding onto the studied polymer substrates. Goniometry showed a change in surface wettability—the polar polymer foil was more wettable, which strongly affected the antimicrobial behavior of the prepared samples. These polymers had very good antimicrobial activity against *D. quadricauda* algae, good antibacterial activity against *S. epidermidis*, and slight against *E. coli*, even at a low cinnamaldehyde concentration.

In this context, designing hierarchically structured and chemically “reactive” durable interfaces is an excellent alternative for tailoring different bio-inspired wettabilities following a common synthetic process. However, designing chemically “reactive” and hierarchically structured interfaces remains a highly challenging task, and the progress of this highly potent research topic has been relatively slow in the literature. UV initiated radical polymerization to develop porous, polymeric, and functionalized surfaces. The top of the hydrophobic surface was functionalized with reactive functional groups to endow the substrate reactivity [92]. Surface treatment using ultraviolet (UV) is an excellent alternative.

#### 3.2.3. Curing Effect

The curing method for conventional curing offers distinct advantages compared to solvent-based coating or thermal curing [93]. In general, photo-polymerization is divided into three main categories known as free radical photopolymerization (FRP), cationic photo-polymerization (CP), and the hybrid initiating system (FRCP). In general, three different systems can be designed toward the synthesis of hydrogels using a photopolymerization approach as follows: use of hydrophilic reactive monomers and bifunctional crosslinkers, use of polyfunctional crosslinkers with or without reactive monomers, and use of non-functionalized polymer chains [94].

The strategy was also applied to the synthesis of various copolymers and block copolymers possessing polyester or polyamide and polyvinyl segments with interesting physicochemical properties. Vinyl polymers are a major class of commodity polymers, including polyethylene, polypropylene, polystyrene, poly(vinylchloride), poly(vinylidene chloride), poly(methyl methacrylate), poly(acrylic acid), polyacrylamide, polyacrylonitrile, and poly(vinyl acetate) [95].

Under UV irradiation, the produced surfaces react with alkenes, thiols, disulfides, and epoxides, allowing for easy post-modifications and patterning. UV irradiation of the disulfide surface causes the disulfide bond to dissolve, releasing thiyl radicals that can react with a variety of compounds including alkenes, alkynes, thiols, disulfides, and epoxides [92]. The method involves light-induced ketoenol tautomerization, which results in active monomers with both the diene and dienophile functionalities required for the reaction and therefore polymerization. Surface functionalization created a new type of reactive surface, one in which the reactive disulfide groups are concealed by hydrophobic chains and postmodification is accomplished by replacing the hydrophobic chains with other molecules via the photodisulfide exchange reaction.

To confirm this, Du et. al. [92] modified the photoinduced thiol–disulfide exchange reaction between 1H, 1H, 2H, and 2H perfluorodecanethiol (FDT) disulfide SH surface with several hydrophilic molecules under UV (5 min, 10 mW/cm^2^ at 365 nm). The hydrophobic (WCA, 161 [°] ± 3 [°]) disulfide surface turned hydrophilic (WCA 9–51 [°]) after modification, showing the attachment of hydrophilic moieties. ToF-SIMS measurements on a patterned FDT surface corroborated the disulfide bond consumption on the 4-pentenoic-acid-modified disulfide surface. The disulfide peak was still visible on the FDT surface modified with hydrophilic thiols and disulfides, even though the surfaces became hydrophilic after the alteration. The stability of the surface was examined under long-term UV irradiation (10 mW/cm^2^ at 365 nm). UV irradiation enables the functionalization and patterning of different molecules due to the photoreactivity of the disulfide bond [92]. The disulfide surface was prepared by esterification of a porous poly(hydroxyethyl methacrylate-co-ethylene dimethacrylate) (HEMA-EDMA) surface with bis(2-carboxyethyl)disulfide (CED). The obtained CED-modified disulfide surface was hydrophilic with WCA of 44 [°] ± 2 [^o^]. After 2 min of UV irradiation (260 nm, 7.5 mW cm^−2^) in the presence of dibutyl disulfide (DBD), the static WCA increased to 128 [°] ± 2 [°], indicating modification with the hydrophobic butylsulfide groups. The produced BD surface can again be modified with CED by wetting the surface with a CED solution in DMF and irradiating with UV (260 nm, 7.0 mW cm^−2^) for 2 min, restoring the original hydrophilicity of the surface (static WCA 45 [^o^]). The photoinduced disulfide exchange was demonstrated, and it was repeated 20 times [92].

The advances in photochemistry have enabled material scientists to utilize low-energy, long-wavelength UV light and visible light to initiate the gelation process [96].

Formation of biomedical hydrogels usually requires the presence of cytocompatible photoinitiators, such as Irgacure 2959, Irgacure 1173, Irgacure 819, Irgacure 651, riboflavin, camphorquinone, and eosin Y. Those photo-initiators can absorb specific light at different wavelengths, including UV (250 nm–370 nm), visible blue and purple (405 nm–550 nm), and red light (750 nm–810 nm). UV-light-induced photo-polymerization (200–400 nm) is the most commonly used strategy for the fabrication of hydrogels through the photo-polymerization approach, especially for biomedical purposes. The double-bonded carbons in these groups are highly reactive and promote a free radical chain growth polymerization when they are exposed to photo irradiation. Hu et al. present UV light centered around 365 nm, because this long-wave UV exposure at intensities less than 10 mW/cm^2^ is tolerated well by most cell types for exposure times less than 3–5 min, providing cell viability following photo encapsulation typically greater than 90%. Methacrylated γ-PGA (mPGA) was prepared via crosslinking between γ-PGA and glycidyl methacrylate (GMA) [97].

In thiol-acrylate/ene polymerization, the photo-initiator radical abstracts a hydrogen atom from a thiol and forms a thiyl radical. Polymerization is caused by the presence of the thiyl radicals, and the corresponding thiolacrylate/ene photopolymerization can rapidly occur with a low light intensity. The residual thiol groups remain to be utilized to functionalize post-polymerization of the hydrogel. The process of thiol-acrylate photopolymerization, the competing Michael-type addition reaction between the thiol and acrylates, takes place at the same time, resulting in a mixed-mode polymerization. Furthermore, the residual thiol groups remain to be utilized to functionalize postpolymerization of the hydrogel; polymer postfunctionalization and step-growth polymerization have been suggested, and it is based on the visible light photocatalytic thiol–ene “click” reaction.

Xu et al. [98] present polybutadiene and poly(allyl methacrylates), modified with a large range of functional thiols. The reactions were performed in the presence of p-toluidine (5 mol %) under UV light (λ = 461 nm, 4.8 W). Allyl functionalities, poly(ethyl methacrylate-r-allyl methacrylate) P(EMA-r-AM), was synthesized with 4-cyanopentanoic acid dithiobenzoate (CPADB) as the chain transfer agent. P(EMA-r-AM), with a variety of thiol compounds in the presence of photocatalyst and UV light (blue led), present a different conversion. In the case of βmercaptoethanol (BME) and 1-thioglycerol (Thiogly), the conversions could reach high conversion only after 60 min of light irradiation (99% for BME and 76.1% for Thiogly). Polybutadiene and poly(allyl methacrylates) were successfully modified with a large range of functional thiols in several minutes (typically, in 20 min). The N-methyl-2-pyrrolidone was used as the solvent and p-toluidine as redox mediator. This approach could be an alternative for UV-promoted radical thiolene chemistry.

Truong et al. [99] studied photochemical cross-linking, leading to the preparation of a versatile hydrogel platform from a poly(ethylene glycol) (PEG) precursor containing the anthracene moiety, which could be crosslinked, for the first time, by low-intensity visible light without any catalyst. It was utilized for photochemistry in hydrogel cross-linking, synthesizing different PEG precursors containing anthracene, triazole anthracene, and benzyl triazole anthracene. The anthracene moiety can be conjugated to the PEGamine end-group via ester bond formation, while the electron-rich anthracene group was prepared by copper (I). The poly(ethylene glycol) (PEG) precursor containing the anthracene moiety can be crosslinked by low-intensity visible light without any catalyst. The biorthogonality of the cross-linking with a polymer concentration of 2 wt % (1 mM) by controlling the time of irradiation demonstrated that fast dimerization of anthracene under irradiation of low-intensity visible light (400−500 nm) demonstrates visible-light-induced surface modification. It was applied in rapid PEG cross-linking to form bio-orthogonal hydrogels suitable for cell encapsulation The reaction following recent demonstration of visible light induced surface modification that was subsequently applied in rapid polymer cross-linking to form biorthogonal hydrogels suitable for cell encapsulation. The versatility of this hydrogel system was demonstrated by the incorporation of bioactive gelatin for enhancement of cell attachment and viability as post-modification with a biomacromolecule [99].

Recent studies have already proved that this hydrogel can provide a stable structure by cross-linking with other polymers through its various link sites. Yu et al. [100] combined the UV-crosslinking chemistry with a 3D printing fabrication technique. Two technologies use UV-induced radical polymerization to cure the printed resin and to construct 3D objects either layer-by-layer or in a continuous fashion. They modified alginate with poly(ethylene glycol) diacrylate (PEGDA) and poly(vinyl alcohol) (PVA) to improve the molding properties and cytocompatibility of the resultant hydrogel (PVA dissolved in deionized water, heated at 60 °C, 1 h at 10% weight/volume (*w*/*v*) concentration; alginate/PEGDA hydrogel was created by mixing alginate and PEGDA at a concentration of 10% *w*/*v*, the ratio of I-2959 was the same as alginate/PEGDA/PVA hydrogel containing 10% *w*/*v* sodium alginate).

The modified hydrogel was processed to a 3D scaffold by a bioprinter equipped with four (UV intensity varied between 4 mw/cm^2^ and 1200 mw/cm^2^, and the intensity of 200 mW/cm^2^) longwave (365 nm) UV lamps to provide in situ photo-polymerization during the printing. Researchers reported that the incorporation of PEGDA and PVA not only improved the mechanical properties and scaffolding ability but also increased cell attachment and viability [101].

Low-density polyethylene (LDPE) films with surface thiocarbonyl groups were prepared by reacting the anhydride groups of poly(styrene-co-maleic anhydride) (PSM) brushes previously grafted from LDPE films with the hydroxyl groups of 3-((6-hydroxyhexyl)oxy)-9H-xanthene-9-thione) (HXT), according to Wang et al. [102]. Finally, four common functional alkenes were successfully ligated onto the surface of LDPE films under visible light at room temperature: poly(ethylene glycol) methyl ethermethacrylate, 2-(perflurooctyl)ethyl methacrylate, 2,3-dibromopropyl acrylate, and diethyl vinylphosphonate. UV-induced surface grafting polymerization produces LDPEPSM films. The polymerization was carried out for various irradiation times, and the WCA was reduced to (93 [°] ± 5 [°]) from (102 [°] ± 2 [°]) and 94.5 [°] ± 4.2 [°] for pure LDPE films. To obtain LDPE-PSM films, a 2.5 min irradiation duration was used [102].

Xia [103] described the UV-curing process as having characteristics of rapid solidification and high activity. Polyethers, such as poly(tetramethylene ether glycol) (PTMG), and polypropylene glycol (PPG) terminated with hydroxy groups, are important materials for toughening epoxy resins. Hyperbranched or linear polymers with hydroxy groups were introduced as active toughening components into the crosslinking system using a chain transfer reaction, as well as the kinetics of the UV-curing process. The light intensity on the surface of samples was 30 mW/cm^2^. The UV-curing process curve can be divided into two regions. The first region, from 0 to about 50 s, represents autoacceleration where the conversion of epoxy groups in the mixture increased rapidly and the second region corresponds to autodeceleration, where the conversion of epoxy groups stabilized, whose effect could be attributed to the terminally reactive OH. After UV irradiation for 600 s, the epoxy group conversion of CE (epoxy resin, 3,4-epoxycyclohexylmethyl-3′,4′-epoxycyclohexyl carboxylate) reached 66.8%. After modification, this value increased to 70.9% and 80.0%. However, it decreased to 78.8%. UV-cured films with or without modifiers were not completely cured after exposure to UV light for 600 s because of a vitrification effect. The UV-cured films undergo plastic deformation more easily, absorbing much more energy when impacted. PTMG can also produce a relatively significant toughening effect, except for the reduced crosslinking density caused by chain transfer reactions, because of its excellent molecular flexibility. A homogeneous appearance without cavities on the fracture surface was observed in SEM analysis.

Dziemidowicz et al. [104] reported post-fabrication functionalization of electrospun PCL fibers using photo-assisted perfluorophenyl azide-N-hydroxysuccinimide (NHS) PFPA-NHSA (solution of PFPA-NHS (20 mg/mL in methanol, 10 μL) was pipetted onto the surface of a PCL fiber mat (0.5 cm × 0.5 cm)). Afterwards, the fiber mats were placed under a UV lamp (8 W, 254 nm, 50 Hz) and irradiated from a distance of 10 cm. The length of exposure was that needed to achieve post-fabrication functionalization using PFPA-NHS. Its characterization was performed to obtain information on the chemical composition of the modified PCL surface. In the PFPA-NHS-functionalized fibers before and after UV treatment of both elements detected, the percentage of nitrogen decreased after UV treatment, which indicated that photolysis of the azide group occurred. The percentage of nitrogen and fluorine present on the surface was relatively low compared to carbon and oxygen of the untreated material, which can be attributed to the polycaprolactone structure making up the bulk of the material. PFPA-NHS-treated fibers before UV exposure incubated in a solution of rhodamine-amine dye showed no fluorescence, while the micrograph of the UV-irradiated sample revealed a homogenous distribution of the dye. On the other hand, albumin-fluorescein isothiocyanate conjugate (FITC-BSA)-treated samples showed some level of fluorescence, with relatively lower intensity observed with blank fibers in comparison with PFPA-functionalized samples before and after UV treatment. This could have been due to non-specific adsorption of BSA to the surface.

#### 3.2.4. UV Degradation Effect

UV radiation causes photooxidative aging, which results in the breakage of polymerchains, producing free radicals and reducing the molecular weight of polymers, resulting in a loss of surface gloss and the significant deterioration of many material properties with time [105]. Most of the synthetic polymers are susceptible to degradation initiated by ultraviolet (UV) and visible light [106]. UV degradation is a surface mechanism that usually affects a thin surface layer with a thickness ranging from a few micrometers to 1 mm at most [107]. This UV energy is enough to initiate the degradation (free radical produce) and is expected to accelerate in the presence of an oxygen-rich environment. The quantum energy from the wavelength of UV radiation can cause microcracks on the surface of degraded polymers [108].

The degradation of the main plastics (nylon, polyethylene (PE), polypropylene (PP) [109], and polyethylene terephthalate (PET)) found in the sea was observed for 6.5 months as they were exposed to UV irradiation [110]. Polyethylene (PE) is resistant to photodegradation due to the lack of chromophores, but the presence of impurities or structural defects in polymers during manufacture or weathering can act as chromophores.

Tang et al. [111] aimed to investigate alteration in morphologies and functional groups of PVC particles exposed to UVB solar radiation in simulated terrestrial and aquatic environment for different periods of time. The morphologies, surface area, pore volume, and pore size of degraded PVC are different from the virgin plastic due to physical change such as the skin of the PVC. Thermal degradation and photodegradation result in new functional groups on the surface of PVC, which changes their physicochemical properties and alters their interactions with contaminants or microorganisms. Textural analysis of SEM images provides information and new hydrophilic functional groups on their surface.

Fairbrother et al. [112] reported on high-density polyethylene (HDPE) that was exposed under controlled laboratory conditions to a range of temperatures (30 °C, 40 °C, 50 °C) and UV light intensities (153 W/m^2^, 61 W/m^2^, 38 W/m^2^, 15 W/m^2^, 8 W/m^2^, and 0 W/m^2^). Photothermal reaction and the UV light dose–damage relationships were determined for the various properties to quantify the effect.

### 3.3. Laser Ablation (LA)

LA of polymers has been studied for a number of years and is beginning to find practical applications. Depending on the chemical composition and physical state of the polymer, a dose of preliminary γ-irradiation can decrease the rate of LA of PVF [113], or it can increase the rate of LA of PE, an ethylene-propylene copolymer [114]; polyamide; and PTFE [115]. It was reported that an increase in the time of laser irradiation leads to an acceleration of the rate of LA for the ablation of polyethylene (PE) [114], poly(vinylidene fluoride) (PVDF), polyamide, and polytetrafluoroethylene (PTFE) [115,116,117,118].

Poly(vinyl alcohol) (PVA) is a synthetic polymer with excellent biocompatibility, biodegradability, and thermal stability, as well as good adhesive properties—these properties make PVA and composites made with PVA useful for a range of technologies including optical and biomedical materials. The study of LA by CO_2_ laser irradiation of PVA was reported by Allayarov et al. The process of LA was conducted in a vacuum chamber with a continuous CO_2_ laser (wavelength = 10.6 μm, power = 40 W, and diameter of beam on the target surface = 9 mm) used. A window of sodium chloride was used to pass the laser beam into the ablation chamber up to 1 Pa. The ablation process was carried out with continuous evacuation, and the vapor droplet ablation products were condensed on aluminum foil placed at a distance of 5 cm from the laser beam incidence spot. The presence of H_2_O enhanced the decomposition rates. This can be attributed to changes in the polymer structure, making it harder to eliminate H_2_O as there are fewer OH groups. Again, as OH groups are lost, the decomposition reactions would slow down. Absorption of radiation from a CO_2_ laser has been studied since just after the development of the laser, having been used in many technologies including materials science for preparing surfaces, as well as in medical applications [119]. CO_2_ laser ablation due to multiphoton absorption, which leads to surface heating, has been demonstrated with many materials, including polymers; radiolysis of PVA leads to the formation of unsaturated bonds. This was confirmed by IR spectrum of the γ-irradiated PVA [119] with the spectrum of the crater formed during its laser ablation showing that all of the bands in the spectrum of the radiolized PVA are also present in the spectrum of its crater formed after LA. Their presence in the spectrum of the ablation crater is associated with both their stability under the action of a laser and the probability of replacing such preradiolysis products with a new portion of them from the PVA that has undergone radiolysis [116].

Poly(dimethylsiloxane) (PDMS) was surface-treated by nanosecond laser irradiation using a Q-switch ultraviolet laser (SLCu-5030i, Strong Laser) with a wavelength of 355 nm. A 10 ns laser pulse with a rate of 30 kHz was reflected on the PDMS surface by a lens with a focal distance of 22 mm. The laser at range of 2.1–3.4 W was controlled by a computer. Laser fluence and scanning speed were the main factors that determined the morphology of the treated surface. The laser fluence levels used in the experiments were set by the laser power levels of 2.3 W, 2.7 W, and 3.1 W. It was found on the treated surface by Raman characterization and was distributed primarily along the edge of the channel and in the sparkling particles as a result of the PDMS decomposition. Micro-cracks dominate the morphology of laser-irradiated PDMS, and their length and orientation are regulated by the laser pulse power and scanning speed [120]. Young’s modulus dramatically increases to about 22.9 MPa, around 10 times that of nature at PDMS [121].

#### UV Excimer Laser

An intense UV excimer laser can trigger chemical reactions to form new polar functional groups [122]. Hence, laser treatment is a rather complicated process when it comes to laser fluence, which may describe the laser and polymer surface in terminology of thermal and photodegradation, ablation, and photooxidation [123]. Because of these favorable characteristics, laser-based techniques have been sought as alternatives to conventional methods to enhance wettability, roughness, and adhesion of the polymers. Although there are several studies on laser-based modification of some polymers, it should be noted that these methods have not been applied extensively to modify the polypropylene surface [124].

PP membranes with 47 mm and 190 μm diameter were treated, with the irradiation of PP membrane consisting of an adjustable exposure mount and MgF_2_ lens of 20 cm focal length. The raw 2.4 × 1 cm beam of the ArF excimer laser (Lambda Physik™, LPX 200) had a wavelength of 193 nm; fluence of 50, 100, 150, and 200 mJ/cm^2^; pulse energy of 0–400 mJ; and pulse duration of 25 ns, at a 1–10 Hz pulse repetition rate. The comparison of the spectra obtained from the reference PP and laser-irradiated sample indicated that the excimer laser irradiation did manage to induce chemical changes on the PP membrane. XPS confirmed the effective performance of the irradiation process on the generation of functional groups, the WCA decrease, which could be attributed to surface morphology. The laser-modified PP membrane by laser fluence of 100 mJ/cm^2^ was increased to its maximum amount. The surface modification of the PP membrane by moderate ArF laser fluence (100 mJ/cm^2^) induced the desired changes without deployment of any environmentally detrimental chemicals [122].

It was reported that a novel “grafting-from” approach can be used to fabricate temperature-responsive membranes using a 248 nm KrF excimer laser [125]. The laser parameters, such as fluence energy/area of 100 mJ/cm^2^, number of pulses *p* = 300, and laser frequency Hz = 1, can be appropriately tuned to fabricate support membrane 30 × 30 mm PET films by excimer laser through a 635-steel mesh (mesh opening = 20 μm) laser ablation in the first step, and pore grafting on the support membranes conducted with 248 nm KrF excimer laser can be achieved by pulsed laser polymerization (PLP) of PNIPAM in the second step. The grafting chamber was closed and purged with nitrogen for 5 min; the sample was irradiated at grafting fluence of 5 mJ/cm^2^ through a quartz window fitted to the chamber, using the same 248 nm KrF excimer laser. The laser frequency was set to either 25 Hz or 100 Hz. It was observed that water permeabilities of over six orders, at room temperature, can be easily achieved after just few seconds of grafting. It was established that low NIPAM concentration in the grafting solution slows down the network formation inside the pores of the support membranes.

The amount of crosslinker majorly affects the temperature responsiveness of the network. Low crosslinker concentration leads to a high degree of temperature responsiveness; however, it also results in a greater number of pulses being required for membrane fabrication. At a higher crosslinker concentration, membranes could be prepared in a relatively shorter period of time at a low number of pulses. The findings of this study provide an alternative approach, coupled with optimizing pulse laser polymerization (PLP) parameters (laser fluence, pulse frequency, and numbers), to tune membrane properties and help us better understand the mechanism of excimer laser grafting on porous membranes. Overall, our dual step process consisting of porous support membrane production coupled with PLP of temperature-responsive hydrogel network in those membranes offers an extremely fast and simple method to prepare stimuli-responsive membranes [126].

Žemaitis et al. also used a picosecond UV laser (λ = 355 nm) to produce riblet surfaces in PTFE samples [117]. CO_2_ lasers offer many advantages as compared to the processing with femtosecond and excimer lasers, with PTFE showing a large absorption to the CO_2_ laser radiation, evolving the absorptivity from 67% at ambient temperature (27.5 °C), up to 98% at 300 °C. Riveiro et al. used the parameters for laser power P (W) (12.5, 15, 17.5, 20.5, 22.5); irradiance I (W/mm^2^) (122.8, 147.4, 171.9, 201.4, 221.0); scanning speed v (mm/s) (150, 275, 400); and spacing between scan lines Δx (mm) 0.031 (90%), 0.0625 (80%), 0.125 (60%). On the PFTE surfaces after laser texturing, the water droplets were dispensed on the surface, which was tilted ≈15. The angle for the water droplets deposited in the laser-treated PTFE surfaces was larger, with the results suggesting the suitable processing conditions required to obtain low wetting surfaces, i.e., a highly amphiphobic PTFE surface. The wettability of the laser-treated PTFE surfaces was substantially reduced, especially for water, where superhydrophobic surfaces were produced. This reduction in wettability is associated with the rough surfaces produced during the CO_2_ laser texturing. Grooves with protruding filaments were observed after the processing. This spongy-like structure has many voids where air is trapped, being responsible for the reduction in wettability, as chemical modifications in the treated areas were not detected [127].

PLLA and other implantable materials (synthetic; thermoplastic; and bioresorbable polymers such as poly(L-lactide) (PLLA), poly (ε-caprolactone) (PCL), poly(glycolic acid) (PGA), or copolymers such as poly (lactic-co-glycolic acid) (PLGA) [128]) are promising as a strategy using pulse sources including UV-generating excimer lasers (193, 248 nm) [129]. Szustakiewicz et al. [130] used an ultrashort pulse fiber laser operating at second harmonic (λ = 515 nm) with a pulse duration of about τ = 450 fs. The PLLA/HA samples (PLLA/HA composite having 10 wt. % of the filler) were modified by scanning the surface laser micromachining system micro Struct vario (3D-Micromac). A femtosecond pulse laser with λ = 515 nm, pulse duration of τ = 450 fs of 3.25 J/cm^2^, pulse spacing time of 10 μm, and pulse repetition rate of 10 kHz was used for surface modification (grooving) of PLLA/HA composite. Regarding the effect of different incubation and extraction times of PLLA/HA (before and after modification) in general, no cytotoxicity was observed for samples. For the incubation time of 24 h, higher viability was observed for PLLA/HA composite compared to non-modified PLLA/HA, while increasing concentration more vitally for cells for PLLA/HA. At 72 h, the results and trend for the PLLA/HA sample were almost identical to that at 24 h. At the same time, proliferation for PLLA/HA was up to 20% higher in comparison to reference material investigated at the same condition. Later, Szustakiewicz et al. [131] reported the modification with laser significantly accelerates the formation of a mineral layer on the surface of PLLA (laser system based on an ArF excimer laser operating at 193 nm, impulse duration was 5–6 ns and a nominal power < 5 W) for laser-activated samples and references, being placed in a plastic beaker filled with simulated body fluid (SBF), then placed in a dryer to 37 °C for specific periods of time (short term: 1 h, 2 h, 4 h, 24 h; long term: 2 weeks, 4 weeks). The modification of poly(L-lactide) with an ArF excimer laser at 193 nm was associated with polymer chain cutting and the formation of additional –COOH carboxyl groups. The research presented a gradual growth of the apatite ceramic mineral layer on the laser-modified PLLA surface, and deposition of the apatite layer on the polymer surface also resulted in a significant improvement in surface wettability (reduction of contact angle even by approximately 30%). The laser modification reduced the molecular weight of PLLA and accelerated the hydrolysis occurring during longer time periods, wherein the laser modification could reduce the time of polymer degradation in the body in potential applications in tissue regeneration.

### 3.4. Electrospinning

The electrospinning process has been considered in terms of the production of microfibers to nanofibers from polymer solutions under high electric field (kV) at atmospheric pressure and room temperature. Electrospinning devices have two main setups, vertical and horizontal. High voltage generates electric charges on polymer solution, and these charges accumulate on its surface. The nanofibers can be formed via the electrospinning process of polymeric materials and their parameters determining the quality of electrospun nanofibers and their applications. These parameters are either polymer properties (concentration, viscosity, surface tension, and conductivity) or processing parameters (electric field, flow rate, needle tip to collector distance) [132]. Because of their high specific surface area and interconnected porosity structure, micro/nanofiber mats have been the focus of extensive investigation [133,134].

**Figure 8:**
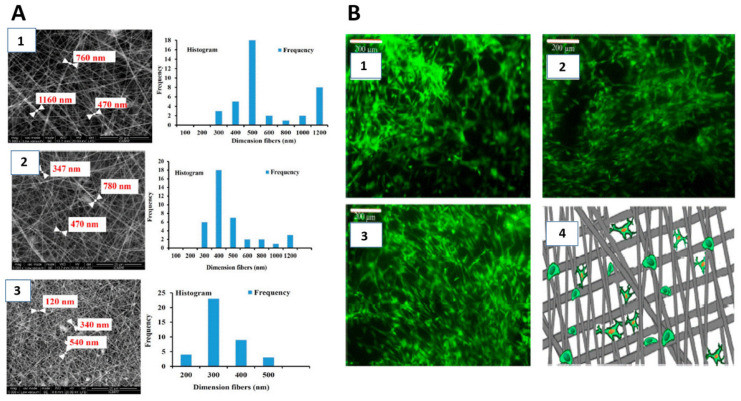
SEM image of the diameter distribution and morphologies of the nanofibrous mats (**A**): (**1**) C, (**2**) C-PET, (**3**) PET. Cells cultured on nanofibrous mats of (**B**): (**1**) C, (**2**) C-PET, and (**3**) PET. (**4**) Schematic mechanism of proliferated cell growth. Reproduced with permission from Ref. [133]. Copyright 2020 Drobota, Gradinaru, Vlad, Bargan, Butnaru, Angheloiu, Aflori under CC BY 4.0.

Figure 8 presents the obtaining of a semisynthetic material by that combining the characteristics of collagen and polyethylene terephthalate (obtained using a new electrospinning configuration) [133]. FTIR-ATR was used to investigate the assembly of the involved macromolecules as well as the factors responsible for the stabilization of the resulting structure, and the diffusion coefficients of the nanofibrous mats from the dynamic vapor sorption–desorption study were displayed in the following order: PET > C-PET > C. The functional groups interacted with water molecules, according to the swelling measurements of the C-PET nanofibrous mat performed in the same study. C-PET nanofibrous mats were found to be non-cytotoxic in a preliminary cytotoxicity testing, indicating that they can be employed in tissue engineering applications.

These distinguishing characteristics, in addition to the inherent capabilities of polymers, endow nanofiber mats with a plethora of desirable traits that make them suitable for a wide range of applications [135]. Nanostructures can be made from a wide range of basic materials, including natural and synthetic polymer composites (both organic and inorganic) [136], and electrospinning is attracting more and more scientists for the highly-efficient preparation of various nanostructures. Wu et al. obtained hierarchical micro/nanofibrous membranes of sustained releasing VEGF for periosteal regeneration by encapsulation of VEGF in a hyaluronan-PLLA core-shell structure. The release was demonstrated in a durable way for angiogenesis in the fibrous layer and the bone defect area [137]. After the PLLA and MS membranes were functionalized, the neutralized collagen solution (50 μL) was dropped on the PLLA and MS membranes, and the samples were introduced in an incubator for 30 min at 37 °C. The regeneration of the periosteum was evaluated by detection of both osteoblasts and periostin, which represented structural and molecular mechanisms. The bone defect was repaired in a uniform and rapid manner by inherent periosteal ossific mechanism involved in both intramembranous and endochondral ossification. The bionic periosteum may represent great promise for clinical treatment of bone defect with periosteal injury.

Liu and coworkers [138] studied fabricated calcium phosphate nanoparticles (CaPs), which were then further incorporated with gelatin-methacryloyl (GelMA) by electrospinning fibers to construct the hybrid hydrogel fibers. The fibers exhibited satisfactory morphology and mechanical properties. A biomimetic composite of CaPs and GelMA hybrid hydrogel electrospinning fiber with the capabilities in promoting osteogenesis differentiation and angiogenesis was successfully prepared. The CaPs with a diameter of 50.7 ± 2.3 nm and zeta potential of 16.4 ± 1.6 mV were fabricated, and then constant ion release was observed for around 14 days from these CaP-capsulated GelMA fibers.

Polysulphone/polyurethane (PSU/PU) nanofibers were fabricated by Chen et al. [139]. The filtration capability was tested against organic and inorganic airborne contaminants. The thick fibers provided mechanical properties, while the thin fibers were used for air filtration. Tissue templates, drug delivery, pharmaceutical components, medical prosthesis, artificial organs, wound dressings, bone tissue engineering, filtration creation, and sensing are just a few of the applications for electrospun nanofibers [140].

Cao et al. [141] studied PET nanofibers prepared from PET dissolved in TFA/CH_2_Cl_2_ (*v*/*v* = 9:1) at room temperature for 12 h, which became a homogeneous solution when the PET solution was 17 *w*/*v* %. The obtained electrospun nanofibrous mats were dried in a vacuum oven at room temperature for 24 h using the following parameters: electric voltage of 25 kV, solution at 4 mL/h. The PET nanofibrous membrane’s porous structure with random fibrous orientation was modified with plasma nitrogen, and parameters were at glow discharge with 150 w, with the time of 180 s. In terms of the modified membranes with acrilyc acid (AA), PET-Q-AA in the low temperature plasma was prepared and immersed in AA aqueous solution. The grafted membrane with carboxyl and acyl amino groups resulted in modified PET nanofibrous membranes possessing a geometrically uniform and defect-free nanofibrous structure and were used for adsorption of Cu^2+^ from aqueous solutions. The adsorption–desorption processes showed the modified membranes can be reusable. With fast adsorption and high adsorption capacity, the PET nanofibrous membrane modified with plasma-induced graft co-polymerization can be considered a potential adsorbent for removing Cu^2+^ from aqueous solution when compared to the capacity of common fibrous filters.

Mohamed et al. investigated the adsorption of arsenic [142] crom ions in water, using composite nanofibers (PAN-CNT/TiO_2_-NH_2_) obtained from electrospun crosslinking of the amino functionalized composite nanofibers (PAN-CNT-NH_2_) to TiO_2_-NH_2_ via the amine side with PAN-CNT-NH_2_. The composite nanofibers showed a strong ability to remove Cr(VI) ions from water, particularly in an acidic environment. The addition of NH2 groups to the TiO_2_ surface can considerably improve the composite nanofibers’ adsorption ability for heavy metal removal [143]. Haddad et al. studied the adsorption of Pb(II) and Cd(II) ions by PAN/TiO_2_ composite electrospun nanofiber. The prepared PAN/TiO_2_ nanofibers had better adsorption capacity for lead and cadmium ions than pure PAN electrospun nanofibers [144].

The membrane fibrous nano/micro-structured PCL/GEL-Has were created by El-Fiq [145]. The novel substance was prepared including electrospinning fabrication and biomimetic mineralization. The PCL/GEL-HAs membrane exhibited a unique nano/micro-structure, bone-mimetic composition, high specific surface area, enhanced wettability, good biodegradability, adequate mechanical strength, and high protein adsorption capacity. Furthermore, the PCL/GEL-HAs membrane showed excellent controlled release of bioactive ions including Ca^2+^, PO_4_^3−^, and SiO_4_^4−^ over 4 weeks. Finally, the membrane sustained the release of cytochrome c (a model protein) for 2 weeks with zero-order release kinetics. Taken together, the PCL/GEL-HAs membrane demonstrated remarkable merits and thus it can be considered a multifunctional bioactive/biodegradable membrane for periodontal bone regeneration.

## 4. Effects of Surface Properties on Biological Responses of the Materials

Surface chemistry, topography, chemical composition, wettability, stiffness, dimensionality, porosity, and degree of cross-linking are fundamental parameters that must be taken into account in the design of substitutes for medical applications. By manipulating surface properties of implants such as surface charge, functional groups, and topography, initial cell attachment can be tuned in a controlled manner [6].

The surface wettability is the adhesive force between the liquid and solid material surface that causes the spreading of the liquid across the solid surface. It is known that proteins tend to bind onto hydrophobic surfaces while cells are typically attached and proliferated on the hydrophilic surface. The surface wettability can be preferentially tuned from hydrophobic to hydrophilic via manipulation of surface chemistry and surface topography [146]. Oxygen content seems to have a high influence in topography, roughness, and contact angle. The cold plasma method is very efficient in sterilizing biomedical materials and offers advantages in comparison with other used methods. Many researchers such as Rodrigues et al. studied the effect of surface wettability degree on fibroblast adhesion [147]. Surface wetting influences cell morphology such as in shape and distribution, which are parameters to be considered for final cell adhesion. Surfaces presenting a more hydrophobic character are shown to favor fibroblast adhesion properties. In applications, excessive fibroblast adhesion may be harmful, and, therefore, a hydrophilic surface is better than a hydrophobic one.

Polymeric film shave has attracted a great interest with numerous potential utilizations, including biomedical application [148]. Much more important is the work regarding the covalent immobilization of the biomolecule on the surface, which is a domain in permanently change, implying the inspiration in creations of technology in various conditions, with optimal functional groups needing to be present on the surface and the best control of the biomolecules immobilized to not alter their properties. It is important for there to be a good agreement between the biomolecules and the functional groups that are present on the surface. When one to two are insufficiently present on the surface, this leads to an inactive surface, and the biological activity is less. The functional groups are an important parameter and the key in multi-anchoring processes. The optimization strategy to produce the biomaterials determines the replacement of the single biomolecule immobilization with multifunctional ways of immobilizing biomolecules. These are reported for functionalization of surfaces in various ways:

(a) the successive immersion of a single surface in different solutions biomolecules containing a mixture of biomolecules [149];
(b) the single immersion of a surface in a solution containing a mixture of biomolecules [150];
(c) the immobilization of synthetic and multifunctional biomolecules, as well as proteins [151].

Solution immersion is one of the easiest preparation methods for superhydrophobic surfaces. According to the substrate material increase or decrease, solution immersion methods are divided into the chemical deposition method and the chemical etching method [152].

Generally, antimicrobial agents are categorized into two classes: organic and inorganic materials. Some examples of the former group are phenols, halogenated compounds, and quaternary ammonium salts, as well as natural materials, such as chitosan and chitin, which have lately been highly regarded [153]. In respect to the inorganic agents, some of the oxidized nanoparticles, including TiO_2_, ZnO [154], MgO, and CaO, have attracted considerable interest as they resist the harsh processing conditions and impose robust biocidal effects against foodborne pathogens.

PET nanofibrous membranes’ porous structure with random fibrous orientation was modified with plasma nitrogen; parameters were at glow discharge with 150 w, with the time of 180 s. In terms of the modified membranes with acrylic acid (AA), PET-Q-AA in the low-temperature plasma was prepared and immersed in AA aqueous solution. The grafted membrane with carboxyl and acrylamino groups resulted in modified PET nanofibrous membranes possessing a geometrically uniform and defect-free nanofibrous structure, being were used for adsorption of Cu^2+^ from aqueous solutions. The adsorption–desorption processes showed the modified membranes can be reusable. With fast adsorption and high adsorption capacity, the PET nanofibrous membrane modified with plasma-induced graft co-polymerization can be considered as a potential adsorbent for removing Cu^2+^ from aqueous solution [141].

Aldea et coworkers [155] produced fibers of poly(methyl methacrylate) by electrospinning and then coated them with a gold layer and attached them on a thin polyethylene terephthalate substrate in order to obtain flexible electrodes for biosensing applications. Another group of researchers reported the obtaining of electrospun fibers from bioabsorbable polymers such as polycaprolactone (PCL) [156], poly(lactic-co-glycolic acid) (PLGA) [157], and poly(lactic acid)/poly(hydroxibutyrate) (PLA-PHB) [158]; natural polymers such as chitosan and gelatin; and blends of bioabsorbable [159] and natural polymers (PCL/PLGA [160] and PCL/chitosan) [161]. In particular, PCL has been extensively studied for drug delivery due to its biodegradability and biocompatibility [162].

### 4.1. Antibacterial Properties

To prevent pathogen infection, the physical and chemical features of the device surface must be altered. The cationic polymers and hydrogels mentioned above can be employed as building materials to create bacteria-resistant coatings for a variety of medical equipment. Surfaces with anti-infective properties include: (1) anti-adhesive surfaces, (2) surfaces with contact antibacterial capabilities, and (3) surfaces with anti-adhesion and contact sterilization properties. Generally, hydrophilicity is unfavorable for microorganism adhesion. On the other hand, grafting with antimicrobial reagents on the surface may realize the contact sterilization [163]. The excellent bactericidal surface was achieved when treated by antiadhesion [164] and bactericidal reagent grafting with the optimized components [82].

The ongoing international pandemic because of COVID-19 has created an awareness of making sure fine practices keep away from the unfolding of microorganisms. In this regard, the studies on growing a floor that destroys or inhibits the adherence of microbial/viral entities has received renewed interest. Although many studies and reviews are focused on antibacterial substances or coatings, there is a distinctly small quantity of information to be found on the use of antiviral substances [165,166]. However, with extra studies geared closer to this area, new statistics are being brought into the literature each day. The mixture of antibacterial and antiviral chemical entities represents a doubtlessly path-breaking intervention to mitigate the unfolding of disease-inflicting agents.

Taking this scenario into account, the development of novel antimicrobial surfaces and biomaterial coatings that can halt microbial contamination and the spread of infection has become increasingly important [166]. In the context of fabrics as a potential source of contamination and infection within the hospital environment, new antimicrobial fiber technologies have emerged. A new fabric has been infused with gallium liquid metal copper alloy particles, showing promising antimicrobial activity against bacteria (antibacterial), fungi (antifungal), and viruses (antiviral) [167]. Development of such fabrics can change the result in the battle with SAR-CoV-2 with respect to personal protective equipment for healthcare workers (i.e., coats, masks, and uniforms), as well as in bed/bath linens and gowns for patients. Nanofiber membranes are ideal for mimicking the native extracellular matrix (ECM), which helps in the regeneration of different tissues. The potential applications of nanofibers are limited, and the process of electrospinning as the main fabrication method still requires development and improvement; hence, new configurations and strategies are continuously being conducted, resulting in unimproved nanofiber membrane properties and functionalities. For a long time, heavy metals have been utilized to combat microorganisms. The most utilized are silver, gold, copper, and zinc. Silver is the most extensively used metal due to its antibacterial properties and low toxicity. Other metals, such as gold, copper, and zinc, have their own antibacterial properties and spectrums [168].

The simultaneous electrospinning of two polymer solutions—PU and AgNP-decorated PEO—has been reported. Via one-step electrospinning of two solutions positioned side-by-side, a hybrid nanofiber membrane was formed, providing antibacterial properties due to the presence of in situ synthesized AgNPs and mechanical strength provided by the PU nanofiber.

Schwibbert et al. [169] tested the colonization of PE surfaces by bacteria after laser treatment. The femtosecond laser treatment adds a nanoscale roughness to the PE surface, enhancing its hydrophobicity (the contact angle of water after the laser treatment goes from 65 [°] to 120 [°]). Both bacterial strains stuck extensively to the pure, hydrophilic areas, whereas the laser-structured, hydrophobic portions showed changes in surface hydrophobicity that could influence bacterial colonization. The findings showed that nanostructures have no effect on *S. aureus* coverage but do lower *E. coli* adherence. Controlling the colonization of polyethylene surfaces by bacteria was obtained by using femtosecond laser surface structuring.

Metal nanoparticle (NP)-containing hydrogels were created by embedding metal nanoparticles in the molecular network of hydrogels. Metal NPs are well known for being easily oxidized, moistened, and agglomerated in the air. All of these flaws will be eliminated once they are placed into the hydrogel matrix. Silver (Ag) and zinc oxide (ZnO) NPs are the most often used metals because these two metals may directly penetrate bacterial cells and bind DNA or oxidative metabolic enzymes, causing the bacteria’s metabolism to be hampered or the DNA to be mutated. Both the hydrogels are good bactericidal materials against *E. coli* and *S. aureus* [170,171]. The in situ synthesis of ZnO nanorods in the crosslinked carboxymethyl chitosan (CMCh) matrix produces a crosslinked carboxymethyl chitosan (CMCh)/ZnO hydrogel that is efficient against *E. coli* and *S. aureus* [172]. When nanoparticle CuO was employed instead of ZnO in the hydrogel CMCh/CuO, it had a similar bactericidal effect on *E. coli* and *S. aureus* [173].

The electrospinning technique involving the production of nanofibers under the electrical forces from polymer solution/melt has been reported for the production of edible nanofibers and textiles for the food industry and biomedical applications [174]. Different biopolymers including chitosan [175], alginate [176], gelatin [177], electrospun gelatin nanofiber containing peppermint, and chamomile essential oil (EO) have been developed. In addition to improvement in antibacterial, antioxidant, and hydrophobic properties of the gelatin nanofibers by encapsulating EOs, the addition of EOs has been shown to strengthen the films by inducing the rearrangement of the protein network and possible crosslinking of the polymer chain by some compounds present in the EO. Application of the electrospun nanofibrous membrane in food preservation has been reported elsewhere [175]. The handheld electrospinning device was used to produce edible chitosan/polyethylene oxide membrane around food. Porosity and interconnected pores of the scaffolds are among the important factors influencing the rate and quality of new tissue formation [178]. In addition to the effectiveness of edible films/coatings in preventing food spoilage and growth of pathogenic microorganisms on the surfaces, they can protect the food from physical, chemical, and biological deterioration during storage/handling/transportation, thereby enhancing selective movement of water vapor, gas, solute and moisture, mechanical handling properties, nutritional quality, and visual and sensory properties on product surfaces and food product functionality, without changing its original ingredients [179].

Sahariah and Másson [180] presented various synthetic routes for chemical modification of chitosan. Chitosan and its derivatives have been widely studied and applied due to their excellent properties such as biocompatibility and antibacterial activity.

Yang et al. [181] observed the effects. Following the successful infiltration of poly(dopamine) and the addition of hydroxyapatite and carboxymethyl chitosan into the preactivated Ti surface during the dopamine self-polymerization procedure, the effects of poly(dopamine)-modified surface coating on the biological behaviors of human gingival fibroblasts (HGFs) and oral pathogens have been systematically studied for early peri-implant soft tissue integration. The poly(dopamine)-modified surface was proven to be a superior substrate for human gingival fibroblast adhesion, dissemination, and proliferation, according to the findings. Antibacterial activity was positively modulated by the addition of carboxymethyl chitosan. It was confirmed that employing poly(dopamine)-modified titanium implants, which have significant potential in the optimal design of dental implants, can produce a combination of improved soft tissue integration and antibacterial activity.

### 4.2. Biocompatibility Properties

Some functional devices currently in use in clinics are not flawless, especially those that are implanted in the body for an extended period of time or must be reusable. The results of research that focuses on protein or cell levels are not always appropriate for the much more complicated bodily environment, where device biocompatibility is sometimes more important than functionality [82].

Interestingly, the PLA nanofibers with porous topography did not facilitate the platelet adhesion and activation, as the relative hydrophilicity detains the serum protein adsorption while demonstrating excellent antithrombogenicity and negligible hemolysis [182]. As a kind of blood contact material, hemocompatibility is essential for hemodialysis membranes.

Polyhydroxyalkanoates (PHA) and poly(caprolactones) are two classes of biodegradable polymers that are well known (PCL). Poly(3-hydroxybutyric acid), one of the most widely used PHAs, has garnered a lot of attention as a drug carrier or scaffold biomaterial because, when compared to other biodegradable chemically produced polymers such as poly(lactide-co-glycolide) (PLGA), polylactate (PLA), polyglycolate (PGA), and polyethersulfone (PES) [183], it has a large number of advantages, such as remarkable biodegradability and biocompatibility, easier processibility, and controllable retarding properties. The modified membranes could maintain a stable flux under hemodialysis conditions, and a dynamic protein adsorption test was performed. Ji et al. [183] studied poly(glycidyl methacrylate) (PGMA) incorporated into polyethersulfone (PES) membranes via a combination of an in situ crosslinking polymerization and the phase inversion method, and following this, poly(acrylic acid-co-2-acrylanmido-2-methylpropanesulfonic acid) (PAA-AMPS) was covalently coated onto the PES membrane surfaces via an in situ ring-opening reaction between the PAA and PGMA by simply immersing the membranes into the PAA-AMPS solution. The blood clots adhered on the surfaces of PES and PES/GMA membranes showed deformed erythrocytes entrapped in a fibrin mesh. The results indicate that coagulation occurred on these membrane surfaces, while for the PES/GMA/PAA-AMPS membrane, no platelet fibrin network and platelet aggregates were observed on the surface, indicating that the membrane had an excellent anticoagulant effect. The hemocompatibility of the modified membranes was significantly improved, as indicated by the prolongation of the activated partial thromboplastin time and thrombin time of PES-based membranes by 157% and 584%.

When highly hydrophilic, biocompatible, and chemically stable polymers, such as poly(vinyl alcohol) (PVA), are used as stabilizers and dispersing agents with favorable mechanical properties for the emulsification of biopolymers, formulations with improved hydrophilicity and optimized degradation rates result. PLGA/PVA, methoxy poly(ethylene glycol)-PCL, PLGA-poly(ethylene glycol), PLGA poly(ethylene glycol)-PLGA, and magnetic SiO_2_/poly(ethylene glycol) have all been described in the literature as encapsulated chrysin (Chr) [184,185,186]. The novel micro-formulations’ cytotoxic effect was assessed against a epithelial human breast cancer cell line, and its hemolytic potential was determined using human blood compatibility. In combination with Chr, an antioxidant, antiallergic, and anti-inflammatory compound [187], the micro-formulation presents important pharmacological and biochemical properties associated with the prevention of cancer. After being exposed to free Chr, hemoglobin is released from RBCs. The hemolytic statistics are consistent with the hematological parameters, indicating that the MCs used are hemocompatible enough. The bioavailability and biocompatibility of the newly developed microformulations, the physicochemical and morphological characteristics, and their sufficient human blood compatibility and cytotoxic activity against cancer cells suggest that MCs can serve as effective delivery vehicles for bioactive flavonoids, making them ideal micro-platforms for future therapeutic applications against cancer and common blood diseases [188].

Functionalization of scaffolds can be tailored for different design features of a specific product. For example, antithrombogenic agents can be conjugated to the surface of nanofibers to extend the patency of a small diameter vascular graft. Adhesion ligands can be conjugated to the luminal surface of the fibrous conduit to recruit endothelial cell progenitors or pre-seed autologous endothelial cells. An electrospun small diameter vascular graft prepared with a fibrin fiber tube encased in a thin sheath composed of PCL nanofiber membrane provided sufficient suture retention strength, an overall mechanical property, and vascular remodeling, as well as enabling long-term graft survival [189].

### 4.3. Cell Adhension

**Figure 9:**
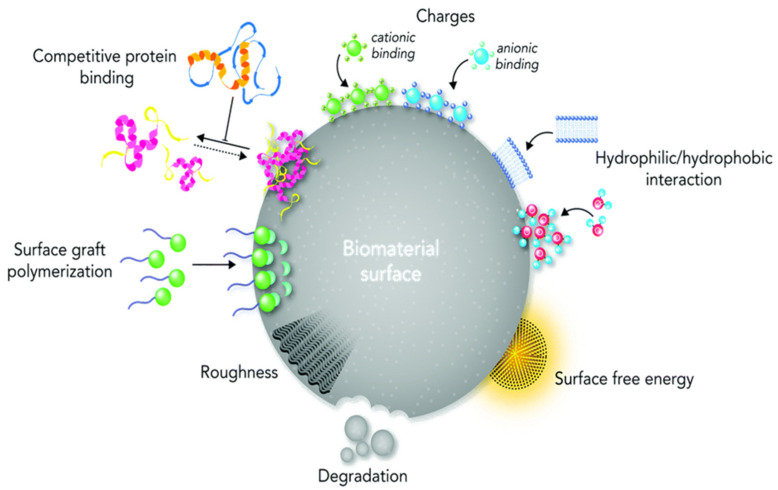
Schematic representation of the main properties responsible for biological responses on the biomaterial surfaces. Reproduced with permission from Ref. [190]. Copyright 2020 The Royal Society of Chemistry.

Biophysical and biochemical signaling pathways involved in cellular responses to the surface can be directed by the physical features of the biomaterial surface. The mechanisms underlying cell responses to these surface features, however, are still unknown. In Figure 9, an illustration of the main physicochemical properties responsible for biological responses on the biomaterial surfaces is presented [189].

After PE plasma treatment a higher BSA concentrations, the adsorption was reduced by over 15%, compared with the untreated membranes. The plasma treatment decreased the hydrophobic interactions between the protein and the membrane surfaces. The lower water contact angle determines the higher level of free water fraction on the membrane surface, which leads to the decrease in BSA adsorption amount on the membrane surface [71].

Aflori and coworkers described the immobilization of collagen molecules on PET after a plasma treatment [68]. The effect of plasma treatment on PET film and further immobilization of chitosan was evaluated in terms of the adhesion and growth of human mesenchymal stem cells. Cell adhesion and proliferation onto PET films after chitosan immobilization depend on the type of substrate pre-treatment before coating. Thus, the cells cultured onto chitosan-coated AC-treated films showed the same morphology as the ones on AC-treated films without chitosan coating. According to MTT assay, the proliferation activity of cells cultured on AC-discharge-treated films was the lowest. In any case, the cells were successfully proliferated on all studied samples and reached confluency at 48 h. However, the formed cell monolayer was easily detached, irrespective of any plasma treatment and chitosan coating [75].

Nanofibers with cells were fixed in 4% paraformaldehyde solution for 30 min at room temperature, and then they were dehydrated with increasing concentrations of ethanol (50%, 70%, 95%, 100%) [78].

**Figure 10:**
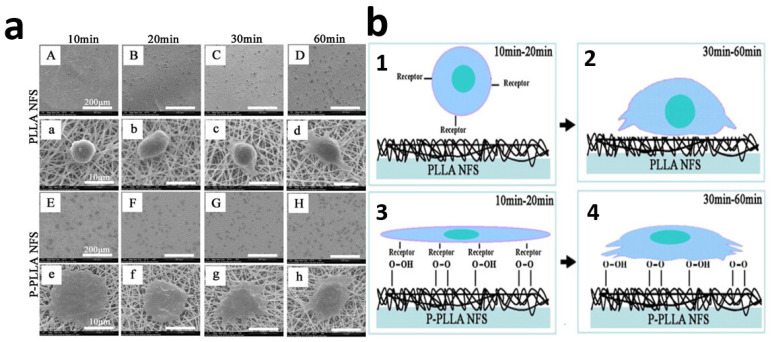
(**a**) SEM images and (**b**) illustration of cell adhesion behaviors on PLLA NFS (**b1**,**b2**) and P-PLLA NFS (**b3**,**b4**) at different time intervals. Reproduced with permission from Ref. [73]. Copyright 2013 Elsevier B.V.

At 10 min, cell capture efficiency on P-PLLA NFS was three times higher than those on PLLA NFS. At 30 min, it was close to 35%, which was five times higher than that at 10 min. A study showed that electrospun nanofibers mimicking natural extracellular matrix corresponded to this study. More importantly, cell capture ability of plasma-treated nanofibers was significantly improved when it was compared with untreated nanofibers. Porcine mesenchymal stem cell (pMSC) attachment behaviors on PLLA NFS and P-PLLANFS were studied at different culture time points (10, 20, 30, and 60 min) [78] (Figure 10), wherein the number of adhered pMSCs on PLLA NFS and P-PLLA NFS increased.

The surface of PLLA NFS was hydrophobic and lacked bioactive groups (Figure 10(b1)). It was difficult for integrin receptors to find suitable sites to bind to when the arrest adhesion occurred. It resulted in the ball-shaped cell morphology at the 20 min interval. As the focal adhesion occurred after 20 min, the cells slightly stretched on the nanofibrous surface, and poor integration between cells and nanofibers was observed (Figure 10(b2)). Plasma treatment introduced oxygen-containing polar groups to the surface of electrospun nanofibers [7]. These oxygen-containing polar groups promoted the hydrophilicity of PLLA NFS and provided enough specific binding sites for integrin receptors to bind to (Figure 10(b3)). Because of the improved hydrophilicity and increased oxygen-containing polar groups on P-PLLA NFS, the cells stretched and integrated well with nanofibrous surface (Figure 10(b4)).

**Figure 11:**
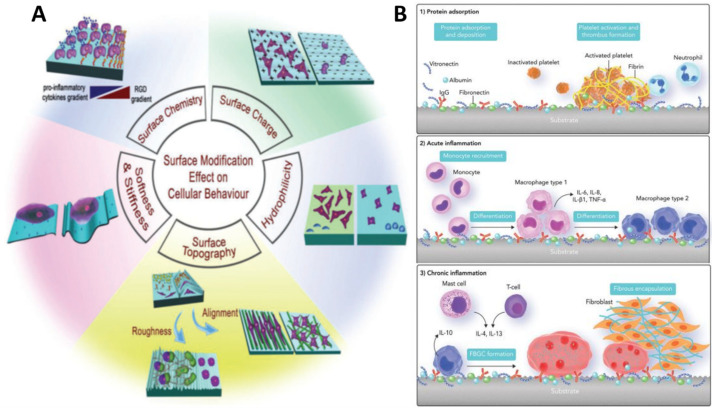
Schematic illustration of surface influence on the biomaterial surface (**A**). The surface properties that may modulate cellular behaviors. Reproduced with permission from Ref. [6]. Copyright 2019 WILEY-VCH Verlag GmbH and Co. KGaA, Weinheim, Germany. (**B**) Traditional theories of foreign body reactions to surface modification. Reproduced with permission from Ref. [190]. Copyright 2020 The Royal Society of Chemistry.

Foreign body reactions are a mix of acute and chronic inflammation. Protein adsorption and desorption (Vroman binding) on the surface of the biomaterial after implantation initiates the action. It continues with the production of thrombin by activating platelets. Monocytes then develop into type 1 macrophages, which are responsible for the acute phase inflammation. Type “1” macrophages develop into type “2” macrophages after a few days, causing chronic inflammation. T cells and mast cells both produce cytokines that promote the formation of foreign body giant cells (FBGCs). Furthermore, FBGCs express fibroblast-recruiting factors, and a capsule form around the biomaterial as a result of collagen deposition [86,190] (Figure 11).

Zhou et al. [191] obtained highly oriented PLLA fiber membranes fabricated via electrospinning, which was treated using the O_2_/He (10^−3^–10^3^ Pa) atmospheric pressure plasma jet (APPJ) with different processing parameters. The surface of the PLLA fiber membranes was functionalized with oxygen-containing functional groups after the APPJ treatment. These oxygen-containing functional groups enable the grafting of gelatin on the surface of the APPJ-treated PLLA membranes. Cell growth on untreated, APPJ-treated, and APPJ-gelatin-treated PLLA-oriented fiber membranes’ surfaces were measured from day 1 until day 7. The O_2_/He APPJ treatment parameters were 1 min and 4 min at 7.5 kV with 0.6% O_2_/He ratio, respectively. Both APPJ and APPJ-gelatin treatments significantly increased the cell proliferation rate due to plasma-induced surface modification, and subsequent gelatin-grafted functionalization was higher than that on APPJ-treated PLLA. The result can be explained by the cell–gelatin interaction being a more integrin-mediated binding. As the treatment time increased, the availability of effective functional groups on the surface increased. As carboxyl groups increase, it would be expected that the interaction between surfaces and cells would be promoted. A similar trend was also observed for the APPJ-gelatin-treated membranes because the increase in the surface functional groups made the gelatin grafting more effective.

Table 1 presents the most relevant methods for modifying surface properties, as well as their main applications.

## 5. Conclusions

It is obvious that chemical and physical sciences are responsible for the current availability of promising biomaterials as therapeutic treatments in medicine. Researchers have made significant progress in the domains of biomaterials and tissue engineering by utilizing natural chemical principles. However, there are still significant biocompatibility issues that need to be investigated further that are sometimes overlooked when discussing new therapeutic options including biomaterials. The biological responses to biomaterials should not be described solely in terms of their lack of negative consequences. Biomaterials can manipulate molecular and cellular signaling pathways through their surface physicochemical properties. In order to drive neo-tissue creation, they must have a substantial affinity for targeted cells, which is dependent on the chemical properties of both biomaterials and biological surroundings. Biochemical signaling pathways and components are primarily responsible for the host’s responses to biomaterials. In this context, surface physicochemistry of a material can profoundly affect biophysical and biochemical responses, being a domain where much remains to be studied.

## Figures and Tables

**Table 1:** The most important methods described in the paper, connecting the surface proprieties with their applications.

No	Methods	Properties	Applications	Reference
1	Chemically hydrolyzed	Increased surface roughness and hydrophilicity antimicrobial wettability	endothelial cell adhesion, hemocompatibility mesenchymal stem cells, osteoblast cell *S. aureus*, *E. coli* human gingival fibroblasts	[11]
				[148,149,168]
				[176]
2	Aminolysis	highest wettability high surface roughness increased hydrophilicity highest sponge structure, surface hydrophilicity increase surface roughness	immobilize bioactive agents such as collagen neural stem-like cells endothelialization cells—vascular grafts, mesenchymal stem cells—regenerative medicine protein adsorption, platelet adhesion—hemodialysis mesenchymal stem cell (MSC) proliferation	[13,14]
				[15]
				[16,19]
				[17]
				[18]
3.	Layer by layer	surface roughness, porosity antimicrobial, biocompatibility	human osteoblasts	[22]
				[42]
				[162]
4.	Surface graft polymerization	hydrophilicity friction coefficient modified topography antibacterial antibacterial, hydrophilicity biocompatibility surface roughness, antimicrobial activity, hemolysis, hemocompatibility	endothelial cell, corneal epithelial cell, MRI contrast imaging artificial hip-joint—osteolysis adsorbtion fibrinogen, human serum albumin, lysozyme and human fibrinogen *S. aureus*, *K. pneumoniae*, *P. aeruginosa*, and *C. albicans* *S. aureus*, *E. coli* fibronectin BSA adsorbtion erythrocyte plasma	[35,41,49,51]
				[43]
				[37,38,39]
				[42]
				[52]
				[54]
				[56]
				[178]
5.	Plasma	wettability, hydrophilicity, topography, morphology, wettability disinfection mechanism, antimicrobial surface wettability	collagen adsorbtion fibroblasts cells, human mesenchymal stem cells, porcine mesenchymal stem cells mouse NIH 3T3 fibroblasts, osteoblast-cells induced bacterial death fibroblast cell fibroblast adhesion L929 cells mesenchymal stem cells	[63,65,66]
				[72]
				[71,73,74]
				[75]
				[82]
				[84]
				[145]
6.	UV	polarity wettability, antimicrobial morphology antimicrobial	*D. quadricauda*, *E. coli*, *S. epidermidis* fibroblasts (3T3), myoblasts (C2C12), endothelial cells human epithelial cell line, skin regenerative osteoblastic cells *P. aeruginosa* (ATCC 27853), *S. epidermidis* (MTCC 435)	[87,122]
				[92,100]
				[147,151,161]
7.	Electrospinning	morphology polarity, antimicrobial biocompatibility porosity, roughness, wettability, higher mechanical properties, hemolysis	human epithelial cell line, skin regenerative human keratinocytes anti-inflammatory activity growth of rat fibroblasts L929 cells stem cells osteoblasts MC3T3-E1 cell fibroblast human osteoblast cell line biological fluids human body plasma red blood cell	[100]
				[123]
				[132]
				[133]
				[134]
				[135]
				[142]
				[146,152,153]
				[169]
				[177]
8.	Laser	roughness, wettability morphology reduction contact angle, higher hydrophilicity antibacterial	human mesenchymal cell differentiation amino acids, simulated body fluid reduced inflammation *S. aureus* and *E. coli*	[121,122,127]
				[129]
				[82]

## Data Availability

The data that support the findings of this study are contained within the article.
